# Harnessing lytic phages for biofilm control in carbapenem-resistant *Klebsiella pneumoniae* causing urinary tract infection

**DOI:** 10.1128/spectrum.03397-25

**Published:** 2026-05-29

**Authors:** Dipendra Kumar Mandal, Elisha Upadhyaya, Puja Dahal, Gaurav Adhikari, Rojina Pandey, Abdul Rehaman Miya, Sudip Timilsina, Pragya Sapkota, Shobha Amagain, David Pun, Kundan Khadka, Keshab Gorathoki, Bijita Neupane, Sangharsika Chaudhary, Sushila Thapa, Binod Khadka, Gun Raj Dhungana, Gorkha Raj Giri, Pragati Pradhan, Krishna Das Manandhar, Roshan Nepal, Rajindra Napit, Rajani Malla

**Affiliations:** 1 Central Department of Biotechnology, Tribhuvan University 526127, Kirtipur, Kathmandu, Nepal; 2 Manmohan Memorial Institute of Health Sciences 590070, Kathmandu, Nepal; 3 Provincial Public Health Laboratory, Lumbini Province, Nepal; 4 Center for Molecular Dynamics 669400, Kathmandu, Nepal; 5 Department of ODS and Research, Meharry Medical College 5708, Nashville, Tennessee, USA; 6 Commonwealth Scientific and Industrial Research Organization (CSIRO), Hobart, Australia; 7 School of Medicine, Deakin University 2104, Burwood, Australia; Newcastle University 5994, Newcastle upon Tyne, United Kingdom

**Keywords:** bacteriophage, biofilm, quorum sensing, phage therapy, CRKP, multidrug resistance

## Abstract

*Klebsiella pneumoniae* is an opportunistic pathogen with rising multidrug resistance and biofilm-related infections. Molecular and phage characterization is crucial to understand resistance mechanisms and explore alternative therapies, such as phage therapy. Whole-genome sequencing and antibiotic susceptibility testing were performed on hospital-isolated *K. pneumoniae* (KP6697), followed by multilocus sequence typing (MLST), plasmid replicon analysis, and antimicrobial resistance gene (AMR) profiling using bioinformatics tools. Phages were isolated and characterized by electron microscopy, with assessments of anti-biofilm activity, lytic efficacy, stability, and host range. Phage genomes were sequenced to identify functional genes. The host *K. pneumoniae* (KP6697) was multidrug-resistant, exhibiting resistance to 18 of 22 tested antibiotics. Genome analysis identified it as sequence type 16 (ST16) with 8 plasmid replicons and 23 AMR genes, including *bla*_CTX-M-15_, *bla*_NDM-5_, and *bla*_OXA-181_. Functional annotations revealed extensive metabolic versatility and a rich repertoire of genes for biofilm formation, quorum sensing, secretion systems, and stress response. A lytic phage, Phage_KP6697_Omshanti, was isolated and classified as a *Caudoviricetes* member with a 45.3-kb genome encoding lysis, replication, and structural genes. The phage demonstrated short latency, high burst size, acceptable thermal and pH stability, and moderate host range against multiple CRKP and other bacterial isolates. Importantly, microscopy confirmed its ability to inhibit and degrade biofilms at multiple stages, highlighting its strong therapeutic potential. Lytic Phage_KP6697_Omshanti, with depolymerase and endolysin activity, isolated from carbapenem-resistant KP6697, showed high burst size, biofilm disruption, and had essential genomic traits suggesting its potential use as an anti-CRKP agent.

**IMPORTANCE:**

*Klebsiella pneumoniae* is increasing multidrug resistance and robust biofilm formation pose severe clinical challenges, limiting treatment options. Understanding the molecular basis of its resistance and exploiting bacteriophages with strong biofilm-disrupting properties provide promising alternative therapeutic strategies. This study highlights the isolation and genomic characterization of a lytic phage with potent anti-biofilm activity against carbapenem-resistant *K. pneumoniae*, underscoring its potential in combating resistant infections.

## INTRODUCTION

*Klebsiella pneumoniae* is one of the six challenging pathogens grouped under the ESKAPE (*Enterococcus faecium*, *Staphylococcus aureus*, *Klebsiella pneumoniae*, *Acinetobacter baumannii*, *Pseudomonas aeruginosa*, and *Enterobacter* species) acronym due to their multidrug resistance and clinical relevance (1). Carbapenem-resistant *K. pneumoniae* (CRKP) has emerged globally as a critical threat largely driven by plasmid-mediated carbapenemases genes that facilitate horizontal transfer across bacterial populations. Alarmingly, CRKP strains have begun showing resistance to last-resort antibiotics like polymyxin and tigecycline (2). Although new β-lactam/β-lactamase inhibitor combinations, such as cefatazidime/avibactam, meropenem/vaborbactam, and imipenem/relebactam, have been introduced, therapeutic options remain limited, promoting exploration of alternative strategies, such as bacteriophage therapy.

A critical aspect of CRKP pathogenicity lies in its ability to form robust biofilms on both biotic and abiotic surfaces. Biofilms are structured microbial communities encased in a self-produced extracellular polymeric matrix, providing bacteria with enhanced survival advantages in hostile environments (3, 4). Within healthcare settings, CRKP biofilms colonize medical devices including urinary catheters, central venous lines, endotracheal tubes, and prosthetic implants, creating persistent sources of infection that are notoriously difficult to eradicate (5–7).

The biofilm lifestyle confers multiple advantages to CRKP, including protection from host immune responses, reduced susceptibility to antimicrobial agents, and enhanced horizontal gene transfer capabilities (8, 9). Biofilm-associated bacteria exhibit tolerance to antibiotic concentrations up to 1,000-fold higher than their planktonic counterparts through various mechanisms, including limited drug penetration, altered metabolic states, and the presence of persister cells (10, 11). This phenotypic resistance, combined with genetically encoded resistance mechanisms, creates a formidable barrier to successful treatment of CRKP infections. The molecular machinery underlying biofilm formation in CRKP involves complex regulatory networks controlling adhesion, matrix synthesis, and community development. Key components include polysaccharide synthesis pathways (such as poly-β-1,6-N-acetyl-D-glucosamine production), fimbrial adhesion systems (including type 1 and type 3 fimbriae), and sophisticated two-component regulatory systems that respond to environmental stimuli (3, 12–14). Understanding these biofilm formation mechanisms is crucial for developing targeted therapeutic interventions that can disrupt established biofilm communities.

Bacteriophages (or phages) are viruses that specifically infect bacteria and particularly lytic phages can lyse bacterial cells. Because of its bactericidal property phages are considered as an alternative measures to prevent and control various bacterial infections including CRKP infections (15, 16). Phage therapy, predating antibiotics by nearly a century, fell out of favor with antibiotic proliferation but has recently regained attention within rising antibiotic resistance (17–19). Clinically, phages can be administered systemically or locally and have shown promise against *Enterobacteriaceae* pathogens, with *Escherichia* phage and *Salmonella* phages being well studied (20–22). Applications of phages against *K. pneumoniae* have been documented in burn wounds and diabetic foot ulcers (23, 24). However, data on phage efficacy against clinical CRKP strains remain scarce.

The rising resistance of CRKP, the role of biofilms in persistent infections, and the potential of bacteriophages highlight the need for innovative treatments. This study bridges the gap between CRKP biofilm formation and phage-mediated disruption by analyzing the molecular mechanisms of biofilm development and characterizing a novel lytic bacteriophage, Phage_KP6697_Omshanti, effective against CRKP biofilms. Functional genomic analysis revealed 87 genes involved in biofilm formation, covering structural, regulatory, and metabolic pathways. The phage demonstrated both anti-CRKP activity and biofilm disruption capabilities. This dual approach elucidating biofilm mechanisms while developing targeted phage therapeutics offers a new strategy for combating antibiotic-resistant infections. Identifying biofilm formation pathways reveals therapeutic targets, while characterizing anti-biofilm phages provides immediate clinical applications and informs the design of enhanced phage therapies. This is critical for CRKP infections, often involving treatment-resistant biofilms. The narrow host range of the phage addresses concerns about off-target effects, enhancing treatment efficacy. By combining mechanistic insights with practical therapeutic development, this research provides a foundation for targeted interventions, phage cocktails, and engineered therapies to tackle CRKP biofilm infections, addressing a major challenge in infectious disease medicine.

## RESULTS

### Antibiotic resistance gene profile of the host bacteria

Antibiotic susceptibility of the host bacteria *K. pneumoniae* (KP6697) was assessed using both the Kirby-Bauer disk diffusion method and the Vitek-2 system with the results elaborated in (Table 1). The isolate was resistant to 18 out of the 22 antibiotics tested. Based on resistance to at least one agent in three or more antibiotic class/categories (25), the isolate KP6697 was considered as a multidrug-resistant (MDR) isolate.

**TABLE 1:** Antibiotic susceptibility test of *K. pneumoniae* (KP6697)**^*a*^**

Antibiotics	Antibiotic class	Code	MIC (Vitek-2)	Inhibition zone (mm)	Interpretation
Amoxycillin (10 µg/mL)	Penicillin	AMP	≥32	0	Resistant
Amoxy-clavulanic acid (20/10 μg/mL)	β-lactam combination	AMC	≥32	12	Resistant
Cefoperazone-sulbactam (75/30 µg/mL)		CPZ	≥64	0	Resistant
Piperacillin-tazobactam (100/10 µg/mL)		PIT	≥128	0	Resistant
Cefepime (30 µg/mL)	Cephalosporins	CPM	≥64	0	Resistant
Ceftriaxone (30 µg/mL)		CTR	≥64	17	Resistant
Ceftazidime (14 µg/mL)		CAZ	≥64	0	Resistant
Imipenem (10 µg/mL)	Carbapenems	IMP	≥16	0	Resistant
Meropenem (10 µg/mL)		MEM	≥16	0	Resistant
Ciprofloxacin (5 µg/mL)	Fluoroquinolones/quinolones	CIP	≥4	0	Resistant
Levofloxacin (5 µg/mL)		LE	≥8	10	Resistant
Nalidixic acid (30 µg/mL)		NA	≥32	0	Resistant
Aztreonam (30 µg/mL)	Monobactams	AT	≥64	8	Resistant
Amikacin (30 µg/mL)	Aminoglycosides	AK	≤2	19	Sensitive
Gentamicin (10 µg/mL)		GEN	≥16	0	Resistant
Doxycycline (30 µg/mL)	Tetracyclines/glycylcycline	DO	≥2	14	Resistant
Tigecycline (15 µg/mL)		TGC	≥2	13	Sensitive
Cotrimoxazole (14 µg/mL)	Sulfonamides	COT	≥320	0	Resistant
Nitrofurantoin (300 µg/mL)	Nitrofuran	NIT	12	13	Resistant
Chloramphenicol (30 µg/mL)	Amphenicols	C	≥8	10	Resistant
Colistin (10 µg/mL)	Polymyxins	CL	<0.5	NA	Sensitive
Fosfomycin (200 µg/mL)	Phosphonic acid	FO	<16	18	Sensitive

^*a*^ Interpretations are made according to the CLSI breakpoint tables. NA, not applicable (not tested).

### Detection of metallo-β-lactamase (MBL) production

The KP6697 isolate was confirmed as MBL-positive by disk diffusion and combination disk methods (Fig. S1A). NDM-mediated carbapenem resistance was verified by Bio-Rad CFX96 PCR System targeting the bla_NDM-1_ gene (~621 bp) using in-house primers: NDM For (5′- CGGAATGGCTCATCACGATC-3′) and NDM Rev (5′- GGTTTGGCGATCTGGTTTTC-3′). PCR conditions were: 3 min 30 s at 95°C; 34 cycles of 30 s at 60.4°C and 1 min at 72°C; followed by 5 min at 72°C, with the entire process completed under 2 h (Fig. S1B).

### Molecular characterization of host *K. pneumoniae* (KP6697)

Sequencing yielded 7.30 million raw read pairs (1.53 Gbp). After quality and adapter filtering with fastp, 3.18 million high-quality read pairs (43.6% retention, 478 Mbp) were obtained, with post-filter Q30 ≥ 72.0% and mean read lengths of 143 and 156 bp (R1 and R2, respectively). Estimated median insert size was 95 bp. The whole genome sequencing revealed that the isolate had the genome size of ~4.9 Mbp and based on the MLST typing, the isolate belonged to Sequence Type 16 (ST16) *Klebsiella*. Plasmid prediction identified a total of eight replicons in this isolate. The plasmids present in the isolates were Col440II, ColKP3, IncFIA(HI1), IncFIB(K), IncFII, IncFII, IncR, and IncX3 (Table 2). The analysis performed for the presence of genes for antibiotic resistance revealed the presence of 23 different antibiotic resistance genes (Table 2). The major groups of antibiotics against which the resistance genes were present were beta-lactams (ampicillin), phenicols (chloramphenicol), aminoglycosides (gentamicin, amikacin and streptomycin), sulfonamides (trimethoprim and sulfamethoxazole), macrolides (erythromycin), fluoroquinolones (ciprofloxacin), and rifamycin (rifampicin). The genes involved in antibiotic resistance were *bla*CTX-M-15, *bla*NDM-5, *bla*OXA-181, *bla*SHV-61, *bla*TEM-214 (beta-lactams), *catA1, cmlA1* (phenicols), *aac (3)-Iid, aadA2, ant (3″)-Ia, rmtB* (aminoglycosides), *ere(A), erm(B), mph(A)* (macrolides), *dfrA12, sul1* (sulfonamides), *qnrS1* (fluoroquinolones), *ARR-3* (rifampicin), and *OqxA, OqxA, OqxB, OqxB,* and *qacE* (Table 2). Furthermore, the CheckV analysis detected two prophage sequences with ~100% complete genomes of predicted genome size 39 and 40 Kbp. The prophage sequences were not further analyzed in this research and are included as supplementary data (Table S3).

**TABLE 2:** Molecular characteristics of *Klebsiella pneumoniae* (KP6697) based on ResFinder tool^*a*^

Plasmid or gene	Data type	Predicted phenotype	% Identity	% Overlap	HSP length/Total Length	Start	End	Accession
Col440II	Plasmid	NA	100	99.65	281/282	3,665	3,945	CP023921
ColKP3	Plasmid	NA	100	81.79	229/280	6,178	5,950	JN205800
IncFIA(HI1)	Plasmid	NA	98.45	99.74	387/388	1,432	1,046	JN205800
IncFIB(K)	Plasmid	NA	98.75	100	560/560	5,084	5,643	JN233704
IncFII	Plasmid	NA	98.44	73.56	192/261	192	1	AY458016
IncFII	Plasmid	NA	100	100	261/261	1,815	2,075	AY458016
IncR	Plasmid	NA	100	100	251/251	6,994	7,244	DQ449578
IncX3	Plasmid	NA	100	100	374/374	29,340	29,713	JN247852
*ARR-3*	Resistance	Rifampicin	99.82	100	543/543	68	610	FM207631
*OqxA*	Resistance	Unknown	99.15	99.91	1,175/1,176	40,333	41,507	EU370913
*OqxA*	Resistance	Unknown	99.15	99.91	1,175/1,176	40,333	41,507	EU370913
*OqxB*	Resistance	Unknown	98.89	100	3,153/3,153	41,531	44,683	EU370913
*OqxB*	Resistance	Unknown	98.89	100	3153/3,153	41,531	44,683	EU370913
*aac (3)-IId*	Resistance	Gentamicin	99.88	100	861/861	885	1,745	EU022314
*aadA2*	Resistance	Streptomycin	100	100	792/792	1,035	1,826	JQ364967
*ant (3'')-Ia*	Resistance	Spectinomycin	99.75	82.51	802/972	189	990	X02340
*bla*_CTX-M-15_ (Class A)	Resistance	Ampicillin, ceftriaxone	100	100	876/876	3658	2783	AY044436
*bla*_NDM-5_ (Sub-class B1)	Resistance	Ampicillin, amoxicillin/clavulanic acid, cefoxitin, ceftriaxone, meropenem	100	100	813/813	8984	8172	JN104597
*bla*_OXA-181_ (Class D)	Resistance	Ampicillin	100	100	798/798	1317	520	CM004561
*bla*_SHV-61_ (Class A)	Resistance	Ampicillin	100	89.31	769/861	28,773	28,005	AJ866284
*bla*_TEM-214_ (Class A)	Resistance	Ampicillin	100	87.92	757/861	3,005	2,249	KP050491
*catA1*	Resistance	Chloramphenicol	99.85	100	660/660	1,537	878	V00622
*cmlA1*	Resistance	Chloramphenicol	99.68	100	1,260/1,260	1,212	2,471	M64556
*dfrA12*	Resistance	Trimethoprim	100	100	498/498	130	627	AM040708
*ere(A*)	Resistance	Erythromycin	99.2	100	1,257/1,257	856	2,112	FN396877
*erm(B*)	Resistance	Erythromycin, Azithromycin	98.95	100.26	764/762	3,997	3,234	X66468
*mph(A*)	Resistance	Erythromycin, Azithromycin	99.68	100.11	922/921	3,304	2,383	U36578
*qacE*	Resistance	Unknown [qacE_1_X68232]	100	84.68	282/333	2,663	2,944	X68232
*qnrS1*	Resistance	Ciprofloxacin I/R	100	100	657/657	1,166	1,822	AB187515
*rmtB*	Resistance	Amikacin, Gentamicin, Kanamycin, Streptomycin	100	100	756/756	2,079	1,324	AB103506
*sul1*	Resistance	Sulfisoxazole	100	100	840/840	3,004	3,843	U12338

^*a*^ ResFinder available at https://genepi.food.dtu.dk/resfinder. NA, not applicable.

### Functional annotations and characteristics of bacterial host *K. pneumoniae* (KP6697)

The genomic analysis of *K. pneumoniae* (KP6697) using the KEGG database revealed a highly versatile metabolic repertoire, with complete representation across numerous key pathway modules (Table S1). The *K. pneumoniae* (KP6697) genome encoded fully intact central carbohydrate metabolism pathways, including glycolysis, gluconeogenesis, the citrate cycle, and pentose phosphate pathway, alongside complete carbohydrate degradation (e.g., D-galacturonate and D-glucuronate), glycogen/trehalose biosynthesis, and energy modules such as the reductive citrate cycle and F-type ATPase. Nitrogen and sulfur assimilation/reduction, fatty acid and phospholipid metabolism, *de novo* nucleotide biosynthesis, extensive amino acid pathways (e.g., lysine, histidine), lipopolysaccharide and glycan metabolism, cofactor/vitamin synthesis (e.g., thiamine, riboflavin, heme), terpenoid/polyketide biosynthesis, and xenobiotic degradation (e.g., toluene, benzoate) were all complete. Notably, the isolate harbored robust antibiotic resistance mechanisms, including carbapenem and methicillin resistance modules and multiple multidrug efflux systems (e.g., MexAB-OprM), underscoring its metabolic adaptability, environmental resilience, and significant clinical concern due to broad antimicrobial resistance capabilities.

Several genes were identified as part of extracellular transport and secretion systems, critical for bacterial interaction with their environment. Notably, genes encoding components of the general secretion pathway (GSP) were prevalent, including K02451–K02464 (general secretion pathway proteins B–O), which facilitate the export of proteins across the bacterial membrane. For instance, K02454 (general secretion pathway protein E, encodes an ATP-dependent component [EC:7.4.2.8]), essential for type II secretion system functionality. Additionally, K02504 (*hofB*) and K02505 (*hofC*) encode protein transport proteins, while K02507 (*hofQ*) supports outer membrane protein transport, collectively enabling the secretion of extracellular enzymes and toxins. The presence of K11935 (*pgaA*) and K11937 (*pgaD*), involved in biofilm poly-beta-1,6-N-acetyl-D-glucosamine (PGA) synthesis, underscores the bacterium’s capacity to form robust extracellular matrices, enhancing community stability and resistance.

Further analysis identified multiple regulatory genes, particularly those encoding two-component systems and transcriptional regulators, which orchestrate social behaviors, such as quorum sensing and environmental response. Genes, such as K07659 (*ompR*), K07657 (*phoB*), K07662 (*cpxR*), K07684 (*narL*), K07712 (*glnG*), K07713 (*hydG*), and K07715 (*glrR*), encode response regulators of two-component systems from the OmpR and NtrC families, modulating responses to environmental cues like phosphate availability, nitrogen levels, and osmotic stress (Table S2) (26, 27). Additionally, luxR family transcriptional regulators (K04333, K07782) were detected, with K07782 (*sdiA*) linked to quorum-sensing regulation, suggesting a sophisticated signaling network for coordinating group behaviors. The presence of sigma factors (K03086, K03087, K03092) further indicates fine-tuned regulation of gene expression, with K03086 encoding the primary RNA polymerase sigma factor and K03092 encoding the sigma-54 factor, critical for stress response and nitrogen assimilation.

#### Social genes for quorum sensing and biofilm production

The genomic analysis of the host bacteria *K. pneumoniae* (KP6697) revealed a diverse repertoire of social genes associated with extracellular functions, regulatory mechanisms, and biofilm formation. The identified genes, annotated with their respective KEGG Orthology (KO) definitions and functional roles, are summarized below, highlighting their contributions to bacterial social behavior, environmental adaptation, and pathogenicity in Table S2.

The functional analysis of the identified KO genes in the host bacteria KP6697 revealed a comprehensive molecular machinery involved in biofilm formation and maintenance. There were total of 87 unique KO entries detected in the isolate, representing diverse functional categories critical for biofilm development and regulation. Several genes and pathways directly associated with biofilm formation were identified. Poly-β-1,6-N-acetyl-D-glucosamine (PNAG) synthesis pathway involving K11935 (biofilm PGA synthesis protein PgaA), K11931, and K21478 (poly-β-1,6-N-acetyl-D-glucosamine N-deacetylase) and K11937 (biofilm PGA synthesis protein PgaD) were present, indicating active PNAG biosynthesis machinery. Further, K13650 (MqsR-controlled colanic acid and biofilm protein A) was identified, suggesting involvement in extracellular polysaccharide matrix formation responsible for colanic acid production. Also, significant enrichment of fimbrial genes and adhesion systems was observed. Type 1 fimbriae, K07345 (major type 1 subunit fimbrin/pilin), appeared six times in the data set, indicating high abundance of type 1 fimbrial components. K07348 (minor fimbrial subunit) and K07350 (minor fimbrial subunit) were present, supporting complete fimbrial assembly. Specialized adhesin K21967 (Mat/Ecp fimbriae adhesin) was also identified, representing specialized attachment mechanisms.

Multiple transcriptional regulatory systems controlling biofilm formation were also detected. Quorum sensing regulation, like K07782 (LuxR family transcriptional regulator, quorum-sensing system regulator SdiA), and K04333 (LuxR family transcriptional regulator, csgAB operon transcriptional regulatory protein), were present. Several two-component regulatory systems like K07659 (OmpR), K07657 (PhoB), K07662 (CpxR), K07684 (NarL), K07712 (GlnG), K07713 (HydG), and K07715 (GlrR) were identified. Further, critical protein secretion machinery was also represented like Type II general secretion pathway (complete Gsp machinery from K02451 [GspB] through K02465 [GspS]) was identified enabling secretion of biofilm-associated proteins. Also, the Type VI secretion system like K11907 (VasG protein) was present indicating potential involvement in inter-bacterial competition within biofilms. Further, multiple iron acquisition systems (or iron-siderophore transport components) were detected. These include siderophore transport (K23186, K23187, K23188, and K25111), representing permease and ATP-binding proteins for iron-siderophore uptake, siderophore biosynthesis, K01252 (bifunctional isochorismate lyase/aryl carrier protein) and K08225 (enterobactin exporter), and siderophore reception, K16090 (catecholate siderophore receptor) and K10829 (ferric hydroxamate transport ATP-binding protein). In brief, the functional category distribution comprised genes for biofilm matrix synthesis (8.4% of total KOs), adhesion and fimbriae (12.6% of total KOs), transcriptional regulation (16.8% of total KOs), iron homeostasis (14.7% of total KOs), protein secretion (18.9% of total KOs), cell envelope modification (11.6% of total KOs), and stress response (17.0% of total KOs).

#### Stress response and enzymatic functions

Genes encoding stress response and enzymatic functions were also identified, contributing to bacterial resilience and metabolic versatility. For instance, K04564 (superoxide dismutase, Fe-Mn family [EC:1.15.1.1]) protects against oxidative stress, while K18699 (beta-lactamase class A SHV [EC:3.5.2.6]) confers resistance to beta-lactam antibiotics. Proteases, such as K04772 (DegQ [EC:3.4.21.-]) and K18988 (serine-type D-Ala-D-Ala carboxypeptidase/endopeptidase [EC:3.4.16.4 3.4.21.-]), likely contribute to cell wall remodeling and pathogenicity. Additionally, K07173 (S-ribosylhomocysteine lyase [EC:4.4.1.21]) and K06998 (trans-2,3-dihydro-3-hydroxyanthranilate isomerase [EC:5.3.3.17]) indicate metabolic pathways for quorum-sensing autoinducer synthesis and aromatic compound degradation, respectively.

### Isolation and physical characteristics of phage_KP6697_Omshanti

**Fig 1:**
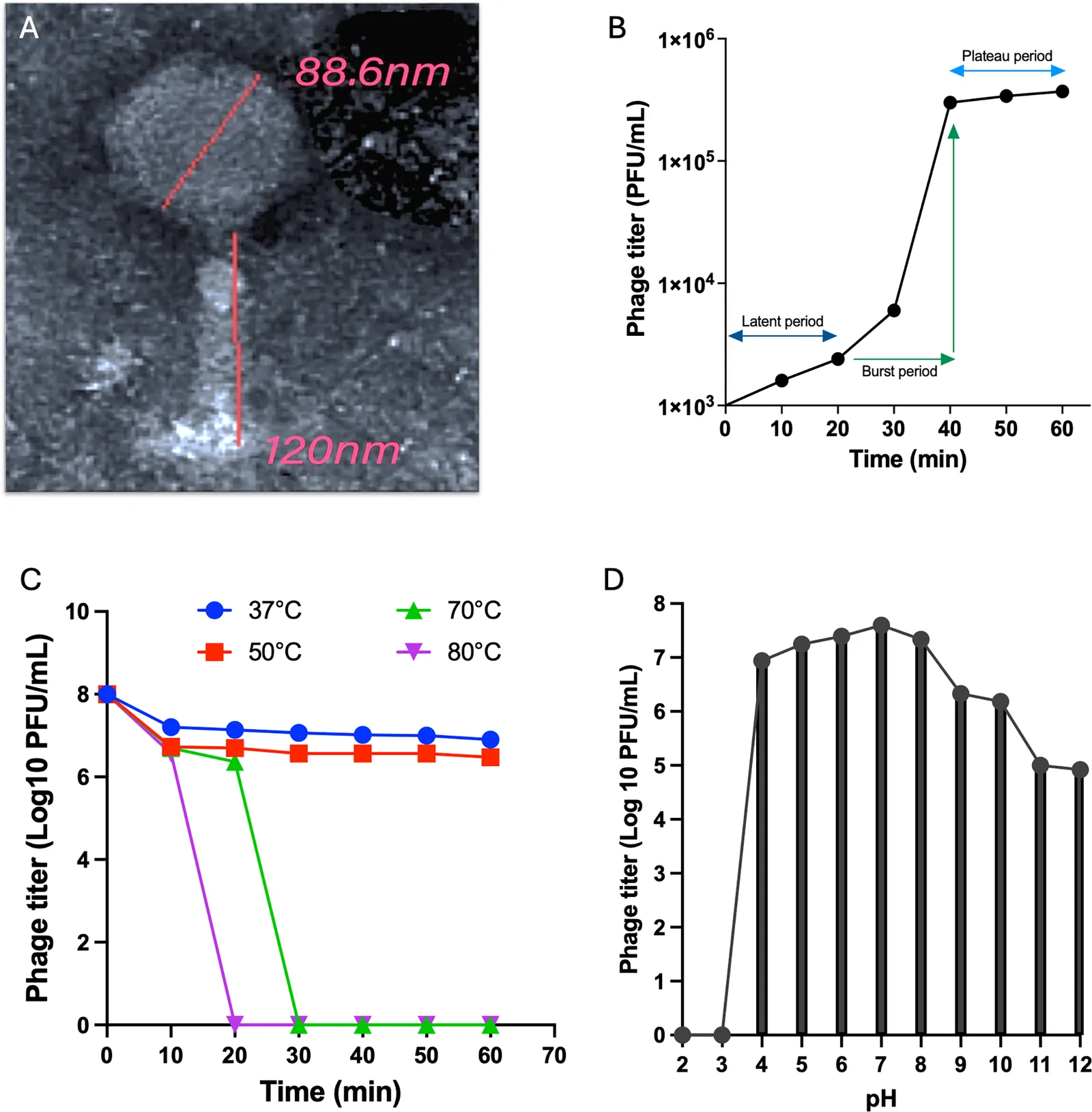
Morphology and characteristics of Phage_KP6697_Omshanti. (**A**) Morphology of Phage_KP6697_Omshanti under transmission electron microscope (TEM). (**B**) One-step growth curve of Phage_KP6697_Omshanti. (**C**) Thermal stability of Phage_KP6697_Omshanti at different temperatures after 1-h treatment. (**D**) Stability of Phage_KP6697_Omshanti at different pH levels after 1-h treatment.

One virulent/lytic phage (hereafter Phage_KP6697_Omshanti) against the bacterial host KP6697 was isolated and purified. The transmission electron microscopy revealed that the Phage_KP6697_Omshanti had an isometric, polyhedral head (88.6 nm in diameter) and a short tail (120 nm long) (Fig. 1A). Phage_KP6697_Omshanti formed opaque halo zones around clear plaques and exhibited a potent lytic activity against the host isolate KP6697. Phage_KP6697_Omshanti formed a bull’s eye like clear plaque (3 mm in diameter) that was surrounded by a large opaque halo zone (Fig. S2B through D).

#### Optimal MOI and one-step growth curve

Based on the maximum phage titer obtained upon infection of host *K. pneumoniae* (KP6697), the optimal MOI of Phage_KP6697_Omshanti was determined to be 0.1. The one-step growth curve of the phage showed a latent period of 20 min and a burst period of 30 min (Fig. 1B), followed by a plateau period. The size of the burst, calculated (28) as the ratio of the average number of phages released per infected host cell before and after the burst, was approximately 101 PFU/cell.

#### Thermal and pH stability

When incubated at various temperatures and pH levels, Phage_KP6697_Omshanti survived steadily over 37°C and 50°C. However, the stability of the phage significantly decreased when incubated at 70°C for 10 min (~2 log_10_ PFU/mL), and upon incubation at 80°C for 20 min, the phage completely lost its stability and viability. These results indicated that the phage could tolerate 37°C (Fig. 1C). The phage maintained good activity in the pH range of 5–9, but its titers dramatically decreased by 1-log and 3-log at pH 4 and 12, respectively. When incubated at pH 3 and 12, the phage was completely inactivated. Thus, the optimum pH range for Phage_KP6697_Omshanti was found to be 5–9 (Fig. 1D).

#### Host range

Phage_KP6697_Omshanti showed a moderate host spectrum on spot assay. Out of a total of 34 strains tested, eight (~23%) were lysed when treated with a single phage, and nine (~26%) were lysed when a cocktail of phages was used (Table 3). Single phage primarily lysed six isolates of *K. pneumoniae*, one *E. coli,* and *Salmonella* Typhi each. However, a cocktail of phages lysed five isolates of *K. pneumoniae*, three *E. coli,* and one *Salmonella* Typhi.

**TABLE 3:** Host range determination for Phage_KP6697_Omshanti^*a*^

SN	Bacterial strain	Isolate source	Spot test	
			Phage_KP6697_Omshanti	Phage_KP6697_Omshanti + Phage 521ø
1	*K. pneumoniae*_115	UTI	+	+
2	*K. pneumoniae*_557	UTI	+	+
3	*K. pneumoniae_*563	UTI	+	+
4	*K. pneumoniae*_9136	UTI	+	+
5	*K. pneumoniae*_9142	UTI	−	–
6	*K. pneumoniae*_6790	UTI	–	–
7	*K. pneumoniae*_9140	UTI	–	–
8	*K. pneumoniae*_207621	UTI	–	–
9	*K. pneumoniae*_8	UTI	–	–
10	*K. pneumoniae*_9	UTI	–	–
11	*K. pneumoniae*_12	UTI	–	–
12	*K. pneumoniae*_13	UTI	–	–
13	*K. pneumoniae*_14	UTI	–	–
14	*K. pneumoniae*_1211	UTI	–	–
15	*K. pneumoniae*_529	UTI	+	–
16	*K. pneumoniae*_531	UTI	+	+
17	*E. coli*_C-19	UTI	+	+
18	*E. coli*_MMIHS	UTI	–	–
19	*E. coli*_C-34	UTI	–	+
20	*E. coli*_1	UTI	−	−
21	*S. aureus*_6739	UTI	−	−
22	*E. coli*_C-48	UTI	−	−
23	*S. aureus*_2994	UTI	−	−
24	*Enterobacter spp*._MMIHS	UTI	−	−
25	*Acinetobacter baumanii*	UTI	−	−
26	*E. coli*_6528	UTI	−	+
27	*Salmonella* Typhi	Blood, Typhoid	+	+
28	*Pseudomonas aeruginosa*_MMIHS	UTI	−	−
29	*Pseudomonas aeruginosa*_6661	UTI	−	−
30	*S. aureus*_6721	UTI	−	−
31	*Citrobacter freundii*	UTI	−	−
32	*E. coli*_9251	UTI	−	−
33	*E. coli*_ESSC	UTI	−	−
34	*E. coli*_ESBL	UTI	−	−

^*a*^ Positive indication (+) means that the strain is susceptible to the phage (i.e., produced clear lytic zone), while a negative indication (−) means that no plaques were observed (i.e., was unable to produce lysis zone). The Phage 521ø (unpublished) is another phage isolated and purified using same host *K. pneumoniae* KP6697.

### Molecular and functional characteristics of Phage_KP6697_Omshanti

**Fig 2:**
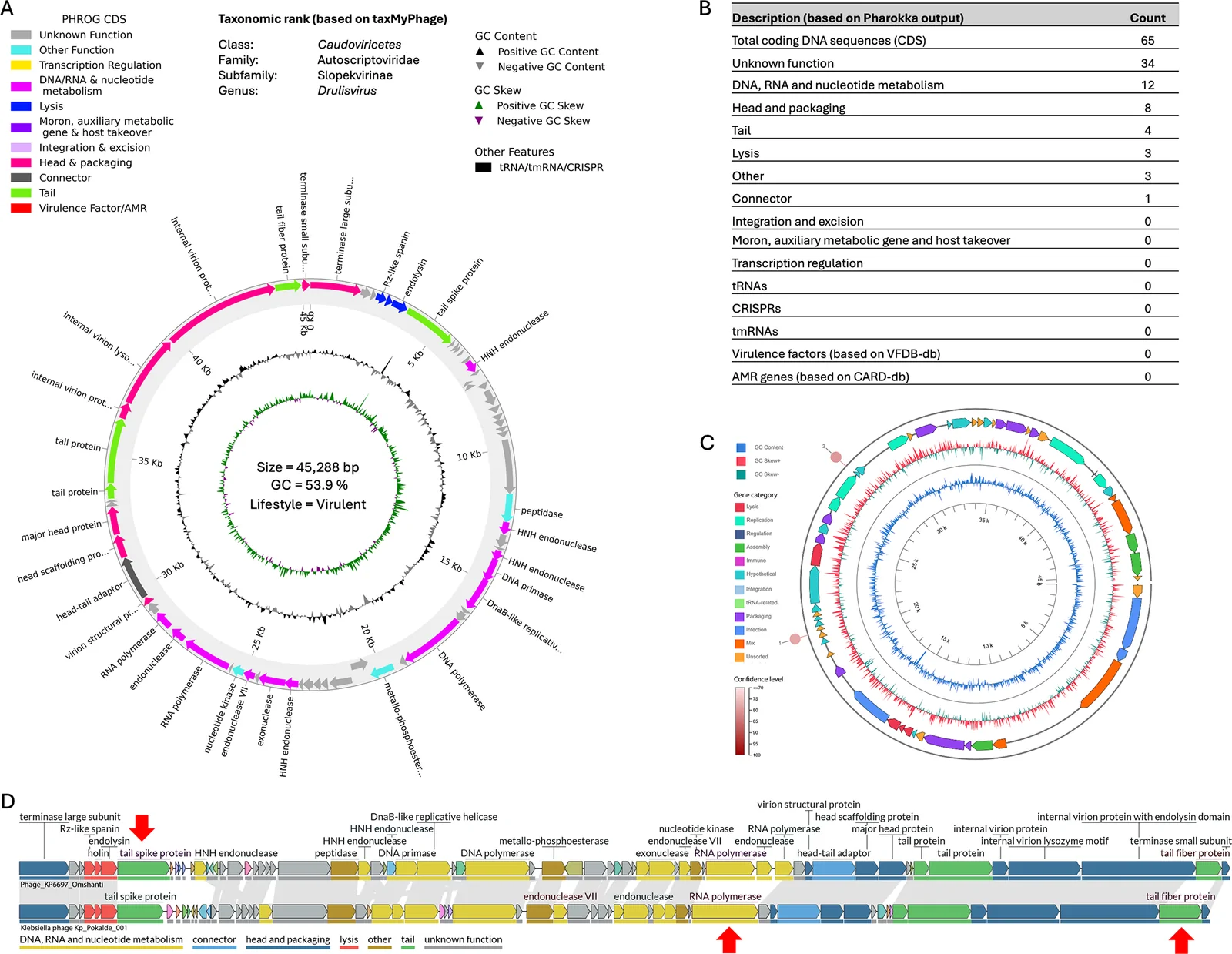
Genome annotation of Phage_KP6697_Omshanti using Pharokka. (**A**) The circular diagram representation of the Phage_KP6697_Omshanti with functional annotation and GC content based on Pharokka output. (**B**) Description of various genes and their number as per Pharokka output. (**C**) Location of two terminase as identified by PhageScope webtool. (**D**) Pairwise alignment between Phage_KP6697_Omshanti and *Klebsiella* phage Kp_Pokalde_001 (GenBank: MW590329.1) isolated from the same river/location about four years apart. The red arrows highlight non-conserved proteins primarily tail spike protein, RNA polymerase, and tail fiber protein.

Whole genome sequencing revealed that the Phage_KP6697_Omshanti belonged to *Caudoviricetes* and had a genome size of 45,288 bp. The phage was most similar to Klebsiella phage RCIP0082 (Query coverage = 81%, Percentage identity = 93.66%, NCBI Accession = OR532876 as of 6 April 2026), which is a lytic phage isolated from sewage water in China using *K. pneumoniae* as a host (Table 4). Phage_KP6697_Omshanti genome was compact with few non-coding sections in between and based on the genomic data the phage has virulent (lytic) lifestyle. The complete genome had a sequencing depth of 916×, with 53.81% GC content and a coding density of 96.88%. Based on Pharokka analysis, Phage_KP6697_Omshanti had a total of 65 genes, of which 12 were assigned to DNA, RNA, and nucleotide metabolism, eight to head and packaging, four to tail (structural), three to lysis function, and a single gene was associated with connector function (Fig. 2A and B). The rest of the genes (34/65) were hypothetical (unknown function) (Fig. 2B) (29). Also, the Phage_KP6697_Omshanti genome contained two transcription terminator sequences (>80% confidence) detected at nucleotide locations 20,567 to 20,586 and 28,023 to 28,041, both in the positive (+ve) strand (Fig. 2C). Phage_KP6697_Omshanti was also similar to Klebsiella phage Kp_Pokalde_001 (query coverage = 79%, percentage identity = 89.81%, NCBI accession = MW590329.1), a lytic phage isolated from the same river around April 2018. Although most of the genomes between Phage_KP6697_Omshanti and Klebsiella phage Kp_Pokalde_001 were conserved, pairwise alignment clearly indicated that the tail fiber protein, tail spike protein (possibly responsible for phage-bacteria interaction), and RNA polymerase were not conserved (Fig. 2D). Further details of Phage_KP6697_Omshanti are elaborated in Table 4 below.

**TABLE 4:** Molecular characteristics of Phage_KP6697_Omshanti based on Pharokka output

Phage name					Phage_KP6697_Omshanti	
Length					45,288 bp	
GC percentage					53.9%	
Coding density					96.9%	
Predicted lifestyle					Virulent (lytic)	
Most similar phage (NCBI)					*Klebsiella* phage RCIP0082 (OR532876)	

Predicted genes, their location, scores and gene functions						
CDS_number	Start	Stop	Frame	Score	Annotation	Gene category
CDS_0001	1	1,857	+	−5.547E+09	Terminase large subunit	Head and packaging
CDS_0002	1,857	2,231	+	−43.604758	Hypothetical protein	Unknown function
CDS_0003	2,243	2,425	+	−9.8902898	Hypothetical protein	Unknown function
CDS_0004	2,425	2,829	+	−47.046031	Rz-like spanin	Lysis
CDS_0005	2,822	3,073	+	−9.3599209	Holin	Lysis
CDS_0006	3,057	3,656	+	−359.11563	Endolysin	Lysis
CDS_0007	3,674	5,632	+	−218,575.24	Tail spike protein	Tail
CDS_0008	5,650	5,754	+	−1.4167005	Hypothetical protein	Unknown function
CDS_0009	5,829	5,969	+	−1.4662557	Hypothetical protein	Unknown function
CDS_0010	5,939	6,028	+	−0.1707603	Hypothetical protein	Unknown function
CDS_0011	6,108	6,206	+	−3.3118295	Hypothetical protein	Unknown function
CDS_0012	6,415	6,564	+	−0.1289323	Hypothetical protein	Unknown function
CDS_0013	6545	7000	+	−36.437714	HNH endonuclease	DNA, RNA, and nucleotide metabolism
CDS_0014	7006	7125	+	−0.0703109	Hypothetical protein	Unknown function
CDS_0015	7241	7122	-	−0.1286163	Hypothetical protein	Unknown function
CDS_0016	7414	7244	-	−0.1075204	Hypothetical protein	Unknown function
CDS_0017	7506	7721	+	−10.735384	Hypothetical protein	Unknown function
CDS_0018	7793	8368	+	−1778.0711	Hypothetical protein	Unknown function
CDS_0019	8434	8679	+	−20.744402	Hypothetical protein	Unknown function
CDS_0020	8742	8951	+	−12.182398	Hypothetical protein	Unknown function
CDS_0021	8944	9207	+	−20.519759	Hypothetical protein	Unknown function
CDS_0022	9216	9395	+	−11.069489	Hypothetical protein	Unknown function
CDS_0023	9392	9598	+	−1.8735946	Hypothetical protein	Unknown function
CDS_0024	9683	11,653	+	−8.244E + 09	Hypothetical protein	Unknown function
CDS_0025	11,653	12,696	+	−494002.09	Peptidase	Other
CDS_0026	12,677	13,144	+	−866.35314	HNH endonuclease	DNA, RNA, and nucleotide metabolism
CDS_0027	13,147	13,620	+	−643.90273	Hypothetical protein	Unknown function
CDS_0028	13,629	13,736	+	−0.2121125	Hypothetical protein	Unknown function
CDS_0029	13,748	14,110	+	−20.610675	HNH endonuclease	DNA, RNA, and nucleotide metabolism
CDS_0030	14,074	14,925	+	−1755.4525	DNA primase	DNA, RNA, and nucleotide metabolism
CDS_0031	14,879	16,135	+	−1937317.3	DnaB-like replicative helicase	DNA, RNA, and nucleotide metabolism
CDS_0032	16,187	16,342	+	−20.619243	Hypothetical protein	Unknown function
CDS_0033	16,339	16,710	+	−1351.8346	Hypothetical protein	Unknown function
CDS_0034	16,685	19,075	+	−2.194E + 13	DNA polymerase	DNA, RNA, and nucleotide metabolism
CDS_0035	19,072	19,293	+	−30.327135	Hypothetical protein	Unknown function
CDS_0036	19,557	20,435	+	−17346.564	Metallo-phosphoesterase	Other
CDS_0037	21,062	20,391	-	−67.759249	Hypothetical protein	Unknown function
CDS_0038	21,133	21,957	+	−2231.0372	Hypothetical protein	Unknown function
CDS_0039	22,010	22,264	+	−16.829522	Hypothetical protein	Unknown function
CDS_0040	22,265	22,543	+	−13.970036	Hypothetical protein	Unknown function
CDS_0041	22,543	22,917	+	−27.853113	Hypothetical protein	Unknown function
CDS_0042	22,920	23,078	+	−3.792375	Hypothetical protein	Unknown function
CDS_0043	23,078	23,584	+	−134.68815	HNH endonuclease	DNA, RNA, and nucleotide metabolism
CDS_0044	23,559	24,533	+	−37286.434	Exonuclease	DNA, RNA, and nucleotide metabolism
CDS_0045	24,514	24,690	+	−15.495117	Hypothetical protein	Unknown function
CDS_0046	24,684	25,106	+	−57.170548	Endonuclease VII	DNA, RNA, and nucleotide metabolism
CDS_0047	25,103	25,546	+	−119.22006	Nucleotide kinase	Other
CDS_0048	25,560	25,649	+	−4.2209054	Hypothetical protein	Unknown function
CDS_0049	25,688	27,502	+	−2.367E + 09	RNA polymerase	DNA, RNA, and nucleotide metabolism
CDS_0050	27,583	28,146	+	−433.77195	Endonuclease	DNA, RNA, and nucleotide metabolism
CDS_0051	28,268	28,942	+	−907.25525	RNA polymerase	DNA, RNA, and nucleotide metabolism
CDS_0052	28,966	29,403	+	−197.6468	Hypothetical protein	Unknown function
CDS_0053	29,403	29,666	+	−5.805937	Virion structural protein	Head and packaging
CDS_0054	29,676	31,271	+	−7256389.7	Head-tail adaptor	Connector
CDS_0055	31,286	32,128	+	−2499.6423	Head scaffolding protein	Head and packaging
CDS_0056	32,154	33,173	+	−52770.516	Major head protein	Head and packaging
CDS_0057	33,185	33,367	+	−6.2741172	Hypothetical protein	Unknown function
CDS_0058	33,339	33,431	+	−4.9953725	Hypothetical protein	Unknown function
CDS_0059	33,464	34,024	+	−1167.3584	Tail protein	Tail
CDS_0060	34,034	36,406	+	−3.23E + 10	Tail protein	Tail
CDS_0061	36,408	36,995	+	−693.56766	Internal virion protein	Head and packaging
CDS_0062	37,012	39,696	+	−9.662E + 12	Internal virion lysozyme motif	Head and packaging
CDS_0063	39,747	44,012	+	−6.35E + 17	Internal virion protein with endolysin	Head and packaging
CDS_0064	44,014	44,970	+	−9302.6174	Tail fiber protein	Tail
CDS_0065	44,987	45,286	+	−109.09807	Terminase small subunit	Head and packaging

Functional annotation of the phage genome revealed that the Phage_KP6697_Omshanti was virulent with only lytic lifestyle. The phage genome was of medium-size based on the genome length (25–100 Kbp) (30) and harbored genes of five different functional categories (Fig. 2B and Table 4). However, there were some unclassified hypothetical/unsorted genes. Furthermore, the genes responsible for replication included proteins, such as DNA polymerase family A, DNA helicase along with two DNA-directed RNA polymerases. These proteins are necessary for phage genome replication and transcription of viral proteins. Another functional category, i.e., packaging of viral particle, included proteins/enzymes, such as three different HNH endonuclease, one endonuclease VII, three endonucleases, and two different proteins with exonuclease activity along with terminase small subunit protein, were present.

**Fig 3:**
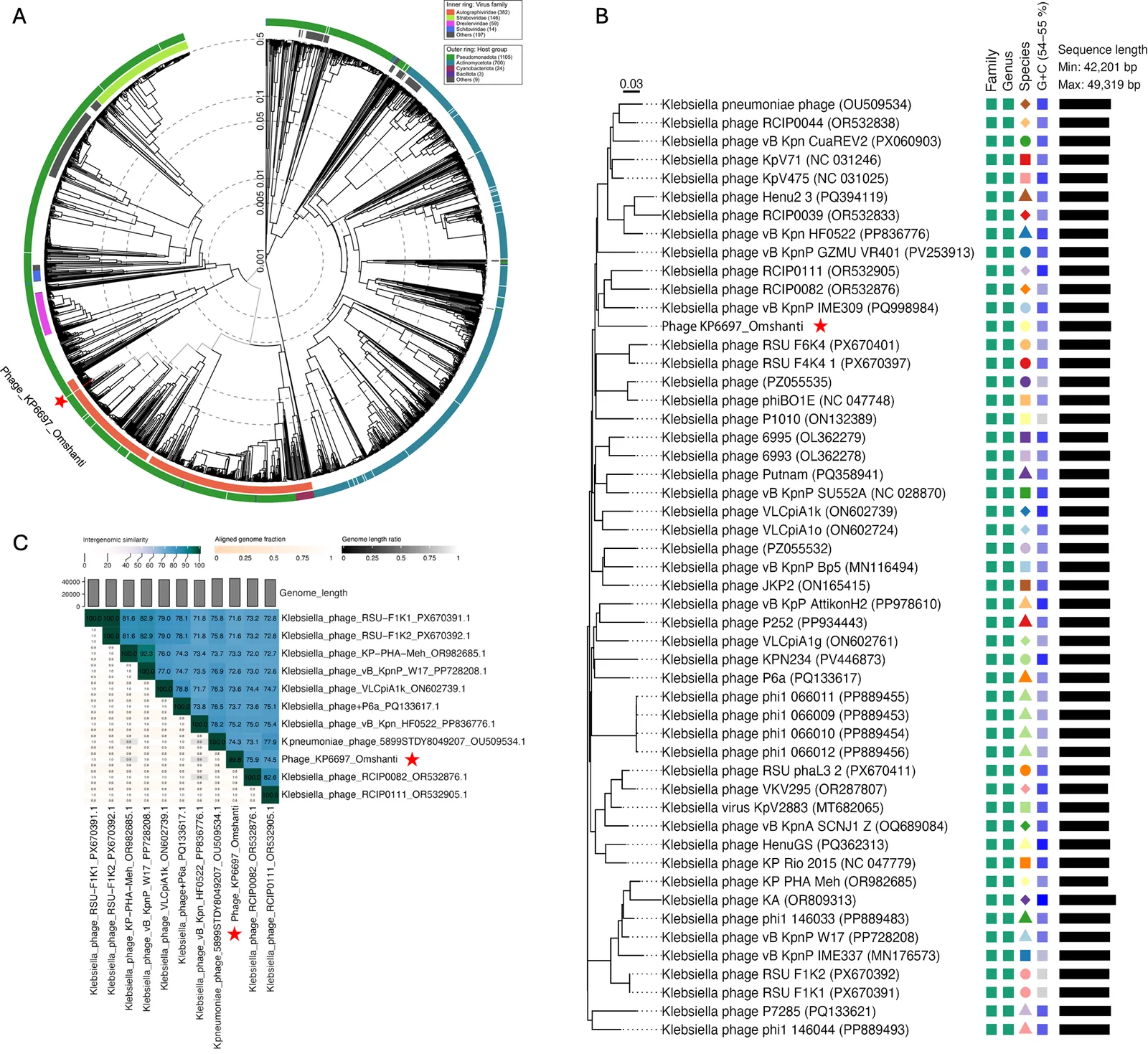
Genome annotation of Phage_KP6697_Omshanti using Pharokka. (**A**) Circular proteomic tree of Phage_KP6697_Omshanti based on ViPTree output with all the “related” genome available in the database. The tree indicates the position of Phage_KP6697_Omshanti based on intergenomic similarities with other related prokaryote-infecting viruses. (**B**) Phylogenetic positioning of Phage_KP6697_Omshanti based on intergenomic similarities with 30 Klebsiella phages (latest genomes) available in the NCBI database. The phylogenetic tree was generated using VICTOR webtool. (**C**) Intergenomic similarity and alignment scores of Phage_KP6697_Omshanti with 10 most-similar hits in the NCBI database. The heatmap was generated using VIRIDIC webtool.

Importantly, structural components that can be used to both infer and validate phage phenotype included head scaffolding protein, viral capsid, virion structural protein, phage head and tail connection and outer membrane proteins. The proteins that are both structural and required for the infection are protein related to tail or tail assembly. Those proteins included tail protein, internal virion protein (part of tail assembly), tail spike and internal virion lysozyme motif proteins were also present. The most significant functional part of the phage that is needed to lyse the cell for phages to be dispersed is the one classified as functional category of lysis. In this category, classic holin, spanin, and lysozyme were present in this phage along with single metallopeptidase like protein. Besides these, the proteins of unknown function and hypothetical protein were also present. Proteome-and genome-based phylogenetic analysis placed Phage_KP6697_Omshanti within *Autographiviridae* virus family and Pseudomonadota host group (Fig. 3A through C), which represents a major phylum of Gram-negative bacteria, corroborating our primary host *K. pneumoniae* (KP6697).

### Effect of Phage_KP6697_Omshanti on biofilm of the host *K. pneumoniae* KP6697

**Fig 4:**
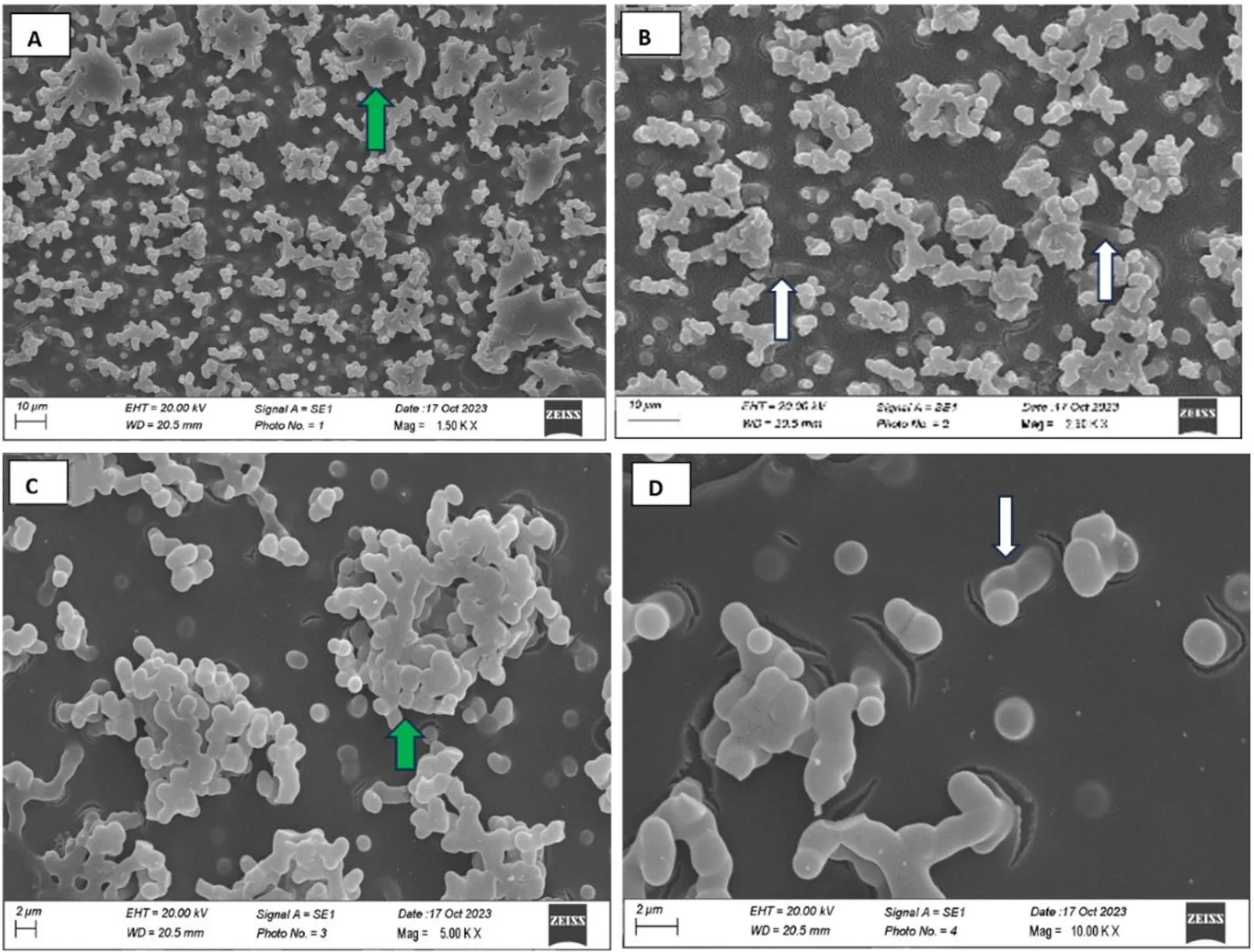
Microcolonies and biofilm of host bacteria *K. pneumoniae* (KP6697) under scanning electron microscopy (SEM) without phage. (**A, C**) Microcolonies (green arrows) of after 24 h of incubation without phage. (**B, D**) Exopolysaccharide connections (white arrows) between bacterial cells after 48 h without phage. A large number of *K. pneumoniae* with intact appearance and high extracellular polysaccharide production are visible.

**Fig 5:**
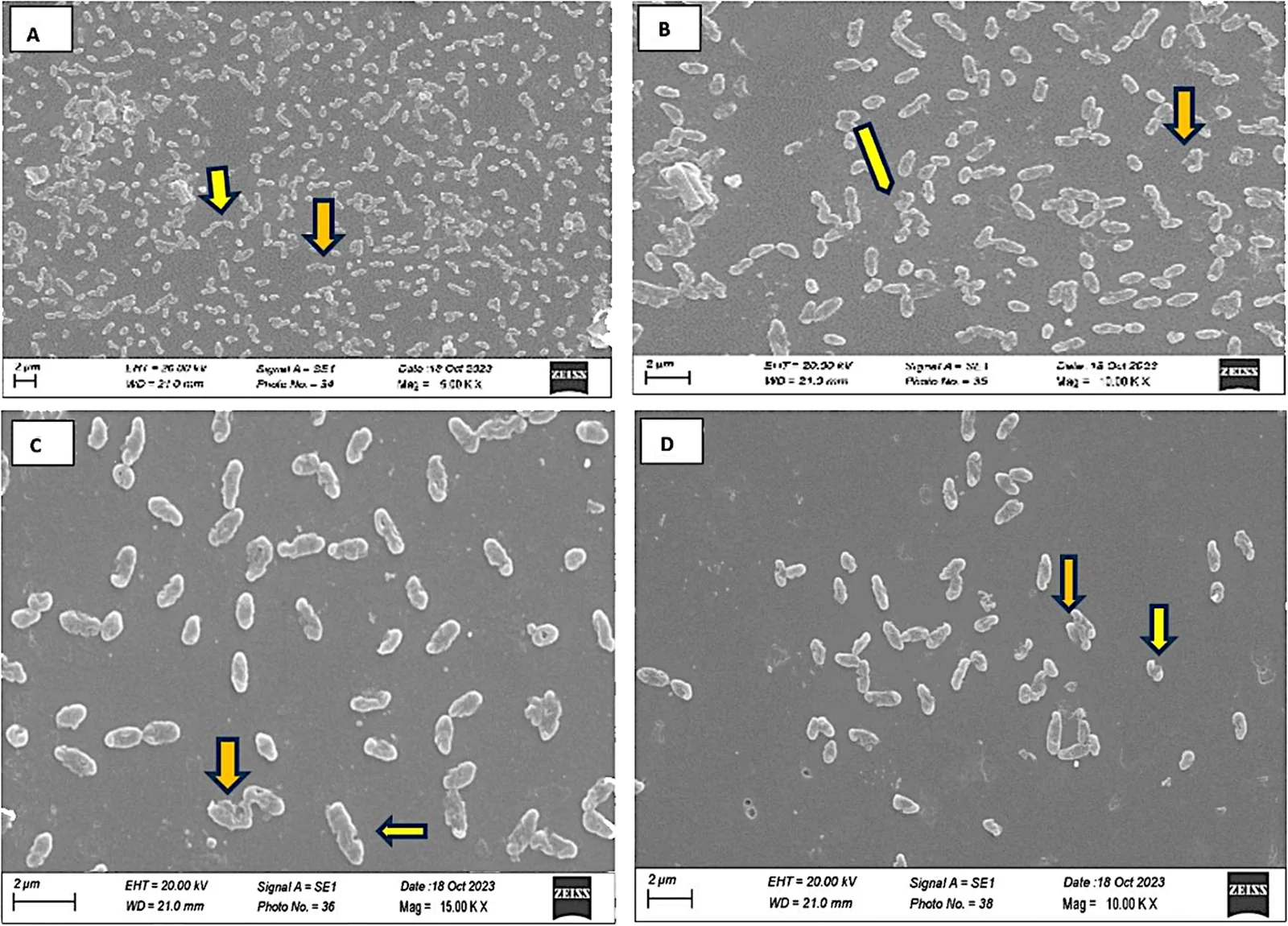
Anti-biofilm activity of Phage_KP6697_Omshanti under scanning electron microscopy (SEM). (**A–D**) *K. pneumoniae* KP6697 treated with Phage_KP6697_Omshanti after 48 h. The micrograph at various magnification shows fewer bacteria, cell wall disruption (orange arrow), forming depressions (yellow arrow) in the configuration of the bacterial skeleton and lessened extracellular matrix.

Under scanning electron microscope (SEM), after 24 and 48 h of incubation, the formation of chains and clusters was observed in control coverslip. While only single cells and small colonies were observed on the experimental coverslip with the addition of single phage. After 24 h of incubation (biofilm formation by KP6697 bacteria), small microcolonies and filaments were observed on the coverslips incubated without phage treatment (Fig. 4A and C), indicating robust bacterial growth. After incubation for additional 24 h (48 h), the formation of mature biofilm with clusters and channels was observed in culture without phage on the surface of coverslips, indicating biofilm formation (Fig. 4B and D). In samples treated with Phage_KP6697_Omshanti alone after incubation for 24 h and subsequent incubation for 48 h, only individual bacterial cells and small aggregates were observed (Fig. 5A through D), indicating inhibition and destruction of bacterial biofilm.

## DISCUSSION

Carbapenem-resistant *K. pneumoniae* (CRKP) strains have emerged as an important multidrug-resistant bacterial pathogens posing a serious threat to global public health. Due to excessive use of antibiotics in hospitalized patients, *K. pneumoniae* exhibits resistance against many antimicrobial drugs. Multiple drug-resistant (MDR) *K. pneumoniae* exhibits several mechanisms, such as the production of extended spectrum β-lactamases and carbapenemases (31). The MDR strains of *K. pneumoniae* cause different infections in humans, which are difficult to control by using antibiotics. Phages are being considered as a potential therapeutic approach against highly drug-resistant pathogens, including CRKP strains (32), and widely proposed as an effective alternative treatment option against multiple drug-resistant pathogens. In this study, a lytic/virulent Phage_KP6697_Omshanti against *K. pneumoniae* (KP6697) was isolated from a river sample and then characterized for different physiological parameters and the whole genome analysis.

The functional analysis of the host bacteria *K. pneumoniae* (KP6697) revealed a sophisticated genetic network for biofilm formation, with 87 KO entries across structural, regulatory, and metabolic categories (Table S1 and S2). Key matrix synthesis components included the complete PNAG pathway (PgaA, PgaB/C, PgaD), supporting active production of this polysaccharide that is known to enhance biofilm structural integrity and antimicrobial tolerance (33), with abundant PNAG N-deacetylases, indicating matrix remodeling (34). Colanic acid biosynthesis (K13650) may have further contributed to extracellular matrix formation. Adhesion was enriched, particularly type 1 fimbrial genes (K07345 detected six times), highlighting their role in surface attachment and sessile transition (35) alongside specialized adhesins like Mat/Ecp fimbriae (K21967) responsible for niche-specific colonization (36).

Regulatory genes involved quorum sensing regulators (SdiA, K07782; CsgAB operon regulator, K04333) for interspecies communication and curli regulation (37, 38), and multiple two-component systems (OmpR, PhoB, CpxR, NarL, GlnG, HydG, GlrR) sensing nutrient limitation, envelope stress, and redox changes (39–42). Nutrient acquisition featured redundant siderophore systems for iron uptake, aiding growth and competition (43–45). Secretion systems included a complete Type II pathway, possibly facilitating export of enzymes and adhesins (46), and Type VI components (VasG) for interbacterial competition (47).

Stress response and resistance determinants, including β-lactamase genes (K18699), combined with biofilm-specific barriers, such as reduced penetration and persister cells, emphasize the clinical challenges in treating biofilm infections (48). Integrated adhesion, regulation, and stress pathways demonstrate biofilm adaptive resiliences to host defenses and therapies (49–51). Further, genes distributed across matrix synthesis (8.4%), adhesion (12.6%), regulation (16.8%), iron homeostasis (14.7%), secretion (18.9%), cell envelope modification (11.6%), and stress response (17.0%), reflected a balanced and multifaceted system in the host bacteria KP6697. These findings highlight the complexity of biofilm development, revealing potential intervention targets like PNAG synthesis, quorum sensing, siderophore uptake, and secretion systems and explains centrality of biofilm in antimicrobial resistance, chronic infections, and environmental persistence, while also guiding strategies for anti-biofilm therapy.

Phage screening and analysis from a number of environmental samples around Kathmandu, Nepal, using the KP6697 isolate as a host bacterium led to isolation of one lytic phage (Phage_KP6697_Omshanti) from water samples collected from Balkhu river. Phage_KP6697_Omshanti was a tailed phage belonging to *Caudoviricetes*. Although the narrow host spectrum of Phage_KP6697_Omshanti (37.5%) was consistent with most of the published research like phages KP1513 and KP-34 against *K. pneumoniae* (52, 53), the polyvalent character of the phage (infecting different bacterial species) is occasionally reported between evolutionary similar hosts (54). As such, Phage_KP6697_Omshanti was possibly a polyvalent phage as it lysed *E. coli* and *Salmonella* Typhi isolates in addition to its primary host *Klebsiella* (Table 3), indicating cross genus host spectrum, which is a highly desirable character for phage therapy. However, we were unable to elucidate the mechanism for this cross-genus specificity. Physiological characterization showed optimal activity at 37°C, with reduced titers at other temperatures and complete inactivation at 70°C similar to phages of the Kp37.

Host range analysis revealed a narrow but notable specificity. The phage lysed 6 of 16 (37.5%) tested CRKP strains of *K. pneumoniae* and showed limited intergenus activity, lysing 2 of 18 (11.1%) MDR strains from other genera, specifically *E. coli* and *S*. Typhi (Table 3). This indicates strong host specificity within the genus *Klebsiella*, with rare cross-genus lysis. The narrow host range is consistent with previously reported *K. pneumoniae* phages KP1513 and KP-34 (55). Further, because of its activity at alkaline pH (Fig. 1D), it could be used as a therapeutic agent for *K. pneumoniae*-mediated urinary tract infection (UTI) and infected wounds, including impregnation of urinary catheters to inhibit bacterial biofilm, as previously suggested (56). Single-step growth curve analysis demonstrated a short latency period of ~20 min and a burst size of ~101 virions per infected cell, which are the hallmarks of efficient lytic phages and support its suitability for therapeutic biocontrol applications (57, 58).

Genomic analysis of Phage_KP6697_Omshanti further revealed a comprehensive array of genes enabling efficient bacterial lysis, biofilm disruption, host defenses and progeny production. Key lysis genes include holins, endolysins, Rz-like spanins, and metallopeptidases that synergistically form pores, degrade peptidoglycan and disrupt the outer membrane, facilitating virion release (Fig. 2) and (Table 4) (59). Phage tail spike proteins and depolymerases target bacterial surface structures and degrade the extracellular matrix to facilitate penetration into biofilms (60). Multiple nucleases counteract bacterial restriction-modification systems and degrade regulatory DNA involved in biofilm maintenance, while helicases and DNA polymerases support rapid phage genome replication. Structural and packaging proteins ensure robust virion assembly and sustained infection in biofilm-protected bacteria. Together, these features underscore Phage_KP6697_Omshanti’s potential as a robust therapeutics against biofilm-associated infections (61, 62). Further, despite high genomic similarity (highly conserved) between Phage_KP6697_Omshanti and another phage Klebsiella phage Kp_Pokalde_001 isolated 4 years prior from the same location, some genes responsible for tail spike protein, tail fiber protein, and RNA polymerase were not conserved (Fig. 2D) indicating evolution of phage.

Unlike studies showing antibiofilm efficacy between single phages and cocktails (e.g., on *Proteus mirabilis* [63]) and *P. aeruginosa* (64), our work focused on a single phage. Phage_KP6697_Omshanti, possessing depolymerase activity, effectively inhibited biofilm formation (limiting development to single cells and small microcolonies) and degraded mature biofilms (confirmed by SEM; Fig. 4 and Fig. 5). Filament formation, linked to biofilm maturation (65) and beta-lactam exposure (66, 67), was observed in control groups but was disrupted by the Phage_KP6697_Omshanti in phage treatment groups. Biofilm reduction was consistent across 24 and 48 h (no significant time-dependent difference in biomass reduction) (68). Mechanisms likely involve capsule depolymerases (69, 70) and holin/endolysin activity (71, 72); lysin genes. As discussed in the results, complete eradication of biofilm was not achieved, consistent with persisters, transient resistance, quorum sensing, and phenotypic heterogeneity in the biofilm (73–76). However, significant bacterial reduction and biofilm destruction occurred and this substantial clearance may suffice clinically, given *Klebsiella* commensal status in the human microbiome (77). Although not attempted in this particular study, phage-antibiotic synergy offers a pathway to full eradication when required, as demonstrated in a clinical case of pan-drug-resistant *K. pneumoniae* (78, 79). The viral RNA polymerase-related elements, though their specific role here is not detailed; such enzymes generally support efficient transcription and are leveraged in biotechnology (e.g., T7/SP6 for mRNA synthesis; 80–83). Structural proteins (head scaffolding, capsid, tail components, tail spikes, internal virion lysozymes) underpin virion stability, host recognition, attachment, enzymatic surface degradation, and genome delivery (84–88), providing a foundation for phage engineering to enhance therapeutic efficacy against antibiotic-resistant bacteria.

Overall, Phage_KP6697_Omshanti stands out for its potent single-phage disruption of CRKP biofilms, making it a promising candidate for anti-CRKP therapy, potentially enhanced by combi nation approaches or depolymerase/lysin engineering. Although complete eradication of bacterial populations was not achieved, significant reductions in biofilm biomass were observed, suggesting that phage therapy, alone or in combination with antibiotics, may provide clinically meaningful outcomes. The structural and enzymatic components of Phage_KP6697_Omshanti not only reinforce its therapeutic potential but also expand opportunities for biotechnological applications, such as engineered phages or phage-derived enzymes for combating MDR pathogens.

### Conclusion

Our study highlights the urgent threat posed by carbapenem-resistant *K. pneumoniae* (CRKP), which combines multidrug resistance with robust biofilm formation, complicating treatment and persistence in clinical and environmental settings. The functional genome analysis revealed a complex biofilm-associated network encompassing adhesion, matrix synthesis, regulatory circuits, secretion, and stress response pathways, underscoring the adaptive resilience of CRKP. Against this background, we isolated characterized Phage_KP6697_Omshanti, a lytic phage with strong lytic activity against the CRKP isolate and some other cross-genus MDR hospital isolates. The *in vitro* assay of the phage demonstrated stability under various physiological conditions like pH, acidity and temperatures, a short latency period, and high burst size, all features desirable for therapeutic use. Genomic analysis further revealed lysis and biofilm-targeting genes, including depolymerases and endolysins, supporting its potential as an anti-biofilm agent. Importantly, SEM observations confirmed its ability to inhibit and degrade biofilms at different maturation stages. Collectively, these findings position Phage_KP6697_Omshanti as a promising candidate for developing targeted therapies against CRKP infections. Future work integrating phage-antibiotic synergy, functional characterization of structural and regulatory proteins, and evaluation in *in vivo* models will be critical for translating these insights into effective therapeutic strategies.

## MATERIALS AND METHODS

### Bacterial strain and culture conditions

All *K. pneumoniae* used either as host (*N* = 1) or host range spectrum analysis (*N* = 34) were received from the Sukra Raj Tropical and Infectious Diseases Hospital, Teku, Kathmandu, Nepal. Preliminary identification of the *K. pneumoniae* (KP6697) bacteria was performed via polymerase chain reaction (PCR) amplification of the 16S rRNA gene using universal primers and protocols (89). Later, whole genome sequencing of this bacterium was also performed using the Illumina MiSeq platform (San Diego, CA, USA). Unless specified, this bacterium was cultured and maintained in Luria–Bertani (LB) medium at 37°C on an orbital shaker at 180 rpm. Phosphate-buffered saline (0.1 M Na_2_HPO_4_, 0.15 M NaCl_2_, pH 7.2) was used for dilution and washing of bacterial cells, unless specified otherwise.

### Antimicrobial susceptibility testing

Antimicrobial susceptibility testing (AST) of the host bacteria *K. pneumoniae* (KP6697) was conducted using fully automated Vitek2 Compact System (bioMeriux, France) following the Clinical & Laboratory Standards Institute (CLSI) guidelines and traditional Kirby-Bauer disk-diffusion methods, as described elsewhere (90). Meropenem and imipenem showed ≥16 µg/mL. The 2023 CLSI breakpoints were used to interpret the susceptibility results (91). All the AST experiment were performed in three biological replicates.

### Phenotypic detection for metallo-β-lactamase (MBL)

The initial screening test for the production of metallo-β-lactamase (MBL) was performed using imipenem (IMP, 30 μg) disk (Mast, UK). If the zone of inhibition (ZOI) was ≤27 mm for imipenem, the isolate was considered as a potential MBL producer. The organism was then swabbed on to a Mueller-Hinton agar (MHA, Hi-Media, Mumbai, India) plate and screened for antibiotic sensitivity. The combination disk (CD) method was used to confirm MBL production according to the following protocol (92). Briefly, for the confirmation of MBL-production, IMP alone and in combination with EDTA were used. An increased ZOI of ≥5 mm for either antimicrobial agent in combination with EDTA versus its zone when tested alone confirmed MBL production (93). *K. pneumoniae* ATCC BAA-1706 (1706, negative for carbapenemase production), *K. pneumoniae* ATCC BAA-1705 (1705; serine enzyme [KPC] positive), and *K. pneumoniae* ATCC BAA-2146 (2146; MBL [NDM] enzyme positive) were used for quality control (91).

### Bacterial DNA extraction, sequencing, and genome analysis

The genome of host bacteria KP6697 was extracted utilizing cetyltrimethylammonium bromide (CTAB) method according to established protocol, as described elsewhere (94). Whole-genome sequencing was performed on Illumina MiSeq platform using DNA Prep kit (20060060). The genome sequences were assembled using Spades (v4.2) genome assembler with careful flag. The contigs were then subjected to taxonomic classification with Kraken2 (v2.1.5) (95) for species identification along with CheckV (v1.0.3) (96) for identification and quality assessment of a viral contigs and prophage detection. Following species identification, the reads were mapped against reference sequence (*K. pneumoniae* strain C17KP0052, Accession number: CP052388.1) with Bwa-mem2 (v 2.3) (97), and consensus was generated with SAMtools mpileup (98), followed by VCFtool-consensus (99). The consensus was scanned against pubMLST using GalaxyAustralia server (https://usegalaxy.org.au/). The whole genome was then annotated with bakta (v1.11.2) and functional annotation was carried out with BlastKOALA (v3.1) (100). Furthermore, social gene responsible for biofilm formation and quorum sensing were identified with SOCfinder (v1.4) (101). The plasmid and AMR contigs were analyzed with StarAMR (v0.11.0) that scans genome assemblies against the ResFinder and PlasmidFinder (102, 103) databases searching for AMR genes using Galaxy Australia server (https://usegalaxy.org.au/).

### Phage isolation and purification

Phage isolation was performed by soft agar overlay technique, as described previously (104), using water samples collected from Balkhu river of Kathmandu valley. Before collection, the water was mixed thoroughly, and the sediments were collected with the overlying water from collection sites in a sterile 50-mL Falcon tube between July and September 2022. A completely isolated clear plaques were picked using sterile needle and dissolved in 1.0 mL sodium chloride-magnesium sulfate (SM) buffer (50 mM Tris−HCl [pH 7.5], 100 mM NaCl, 10 mM MgSO_4_, and 0.01% gelatin). The mixture was then filtered through 0.2-μm syringe filter (Axiva Sichem, Haryana, India) to remove the bacterial and agar debris. The filtrate was further used for soft agar overlay assay and next day, an isolated plaque was picked. The process was repeated three times to ensure pure phage strain was collected from the plates of last isolated. For this, the plates from third round containing plaques were flooded with 10.0 mL of SM buffer and 2 drops of chloroform was added into it. The plates were sealed and incubated at rotating shaker (80 rpm for 30 min) for phage elution/diffusion from the plaques. The SM buffer was then collected and centrifuged at 4,000 rpm for 20 min. Then, the supernatant was filtered through 0.2-μm syringe filter (Axiva Sichem, Haryana, India) to obtain high titer of pure phage. The purification, counting, and propagation of phage were performed using the in-house optimized double-layer agar assay, as described previously (105, 106). SM buffer was used for the dilution of the phages. Finally, the phage lysates were stored at either 4°C or at −80°C in glycerol (3:1 [v/v]) (107) until further use.

### Transmission electron microscopy

The phage lysate was fixed with 2% paraformaldehyde and 2.5% glutaraldehyde. Ten microliter phage lysate was spread on a carbon-coated copper grid and negatively stained with 2% (w/v) uranyl acetate (pH 4.5). The copper grid was dried and examined under the FEI Tecnai T-12 Transmission Electron Microscope (TEM) (FEI Company, Hillsboro, OR, USA) at an accelerating voltage of 80 kV. Phage morphology and taxonomy were confirmed following the guidelines from the International Committee on Taxonomy of Viruses (108).

### One-step growth curve and burst size determination

A single-step growth curve was performed following Kim et al. (109) to determine the phage latent period and burst size. In brief, 10 mL exponentially growing host bacteria KP6697 (OD_600_ = 0.08–0.1) was infected with purified phage at an MOI of 0.1 and was allowed to adsorb for 15 min at 37°C. Subsequently, cells were pelleted by centrifugation (12,000 × *g* for 5 min), and unadsorbed phages were removed by washing the pellets with fresh TSB. Cell pellets were then resuspended in 10 mL fresh TSB and incubated at 37°C. Cultures were incubated for 120 min and after every 10 min, a sample 100 µL was taken for double-layer agar plaque assay. Then, 10.0-µL aliquots (10-fold serial dilution if required) were spotted on double-layer agar plates seeded with the host bacteria. The plaque assay was conducted in triplicate. Burst size was calculated using following formulae: burst size = Average of plateau period/average of latent period (110).

### Thermal and pH stability

The stability of isolated phage at different temperatures and pH was determined according to D'Andrea et al. (111) with slight modification. Briefly, known phage lysate (10^8^ PFU/mL) in SM buffer was adjusted to different pHs ranging from 2 to 12. Phage suspensions were incubated for 60 min at 37°C and then titrated using a double-layer agar assay on the primary host. For temperature stability, known phage lysate (10^8^ PFU/mL) was aliquoted into the Eppendorf tube and incubated at (37°C, 50°C, 70°C, and 80°C) for up to 180 min and titrated using double-layer agar assay as described earlier.

### Host range analysis

The host ranges of the purified phage was determined against 34 different multidrug-resistant isolates using spot testing (20), following previously described method (112). In brief, 100 µL of log-phase bacterial culture (OD_600_ = 0.25) was combined with 3.0 mL of semi-solid top agar (TSA 0.5% agar, maintained at 50°C) and promptly poured onto TSA bottom agar plates (1.5% agar). After solidification at room temperature, each plate was divided into five equal sections. Onto the bacterial lawn, 5.0-µL aliquots of 10-fold serial dilutions of the phage lysate (10^8^ PFU/mL) were spotted and allowed to absorb completely. Plates were incubated overnight at 37°C, after which clear lysis and phage plaques were recorded. The plaque assay was performed in triplicate.

### Anti-biofilm activity of the phage

Biofilm of host bacteria KP6697 was established in six-well polystyrene tissue culture microplates to achieve an improved cell attachment. TSB supplemented with 1% D-(+)- glucose was used to perform this assay, as this helps to improve biofilm formation (113). Briefly, an overnight culture was in the microplate wells; a 1:20 dilution was performed by adding 10 µL of the bacterial suspension to 190 µL of TSB. Two hundred microliters of broth was added to a set of wells as a negative control. All wells were replicated three times. Afterward, microplates were incubated at 37°C for 48 h with no shaking for biofilm formation. During the incubation time (24 h after incubation). Then, 100 µL of fresh glucose was added to all control and test wells. Following incubation, medium was poured off, and wells were carefully washed twice with sterile phosphate-buffered saline (PBS) solution to remove any planktonic cells. Micro-plates were allowed to dry for 1 h at 50°C. To determine total biofilm biomass, microplate wells were stained with 0.1% crystal violet (CV). After staining, the wells were washed twice with PBS and dried. Biofilm formation was first visually compared and photographed. For quantitative analysis, optical density readings of the staining intensity, 10 µL of 95% (v/v) ethanol was added to each well, and optical density at 590 nm (OD_590_) was measured using a plate reader. After 24 h of biofilm formation, 0.1 mL of phage with a concentration of 10^8^ PFU/mL of a single phage was added in six-well polystyrene tissue culture microplates and incubated for 48 h at 37°C. Glass coverslips were added in all wells with and without phage addition. For optical microscope visualization, coverslips were removed with tweezers and placed into petri dishes with paper filters at the bottom without drying. To preserve the natural form of biofilms, samples were vapor-fixed with 25% glutaric aldehyde for 3 h. After fixation, the samples were stained with DAPI (Sigma-Aldrich, Germany). The effect of phage on biofilm formation and destruction was evaluated using an AxioImager A1 light microscope (Carl Zeiss, Germany). After 24 h of biofilm formation, a single phage was added to the biofilm and incubated for additional 24 h. Control was also incubated for 72 h without the addition of phage.

### Scanning electron microscopy (SEM)

Few samples of biofilm producing host bacteria KP6697 treated with phage were fixed with 2.5% glutaraldehyde for 60 min, then dehydrated in a graded ethanol series (30, 50, 70, 80, and 96%) and placed into acetone. The samples were dried in a critical-point dryer HCP-2 (Hitachi Ltd., Japan) and coated with Au–Pd in IB-3 ion coater (Eiko Engineering Co., Tokyo, Japan). Samples were visualized in Camscan-S2 (Cambridge, UK) scanning electron microscope (114).

### Phage DNA extraction, sequencing and genome analysis

The genome of isolated phage was extracted utilizing phage DNA extraction kit (Norgen Biotek, Canada, Cat No.; 46800) according to manufacturer’s protocol. Supernatant with high concentration of phage was used to isolate the genomic DNA. Phage whole-genome sequencing was performed using the Illumina Miseq platform using DNA prep kit (20060060). The genome sequence was assembled using Spades (v4.2) genome assembler with default selection without any careful flag. The contigs were then subjected to CheckV(v1.0.3) (96) for identification and quality assessment of a viral contigs. Thus, identified contigs with complete genome assessment from CheckV was then reoriented with DNAapler (v1.2.0) (115) annotated and classified using Pharokka (v1.7.5) (116) and an online bacteriophage tool PhageScope (https://PhageScope.deepomics.org/), which offers comprehensive annotations including host assignment using BLASTN (v2.5.0), taxonomic annotation using HMMsearch, structural annotation using Prodigal (v2.6.3), functional annotation like transcription terminator annotation using TransTermHP (v2.09), virulence factor and antimicrobial resistance using MMseqs homology against CARD and VFDB databases. The annotated genome was then visualized using Pharokka and LoVis4u (v0.1.5) (117). A proteomic-based phylogenetic clustering of Phage_KP6697_Omshanti was done using ViPTree webserver (v4.0) (Fig. S3B) (118). Further, for an updated similarity analysis, Phage_KP6697_Omshanti queried against NCBI Viruses database (taxid:10,239) and 50 most similar *Klebsiella* phages (Table S4) were extracted and phage taxonomy and genome-based phylogeny was inferred using VICTOR web service (https://victor.dsmz.de) (119). Further, the intergenomic similarity between Phage_KP6697_Omshanti and 10 other most similar Klebsiella phages were assessed using VIRIDIC webtool with default species (95%) and genus (70%) threshold (Fig. 3C) (120).

### Bioinformatics, statistical analysis, and data availability

Statistical analysis was performed using Prims (v9.4) (GraphPad Software, USA,) and *P* < 0.05 was considered statistically significant unless stated otherwise. The original results of the study are included in the article. Additional data are included as supplementary data. Further inquiries can be directed to the corresponding author(s). The complete genome sequences of Phage_KP6697_Omshanti have been deposited in GenBank database under the accession number SRX27372467 and SRA ID number 36925110. Submitted: 11/1/2025; Accepted: 11/1/2025; Published online: 16/1/2025. Similarly, the complete genome sequences of *Klebsiella pneumoniae* (KP6697) have also been deposited in GenBank database under accession number SRX26555504 and SRA ID 35877059. Submitted: 16/10/2024; Accepted: 16/10/2024; Published online: 30/10/2024.

### Limitations

This study did not determine the exact mechanism by which Phage_KP6697_Omshanti lysed its host bacteria as it was beyond the project’s scope. Further research is needed to understand its inter-genus lysis capability, which could aid in designing synthetic phages with broader host ranges. Limitations also include testing the phage only against CRKP isolates from a single hospital and the full characterization of only one phage isolate.

## SUPPLEMENTAL MATERIAL

Supplemental material spectrum.03397-25-s0001.pdf Fig. S1 to S3; Tables S1 to S4.

ASM does not own the copyrights to Supplemental Material that may be linked to, or accessed through, an article. The authors have granted ASM a non-exclusive, world-wide license to publish the Supplemental Material files. Please contact the corresponding author directly for reuse.
