# Supplementary material for: Harnessing lytic phages for biofilm control in carbapenem-resistant Klebsiella pneumoniae causing urinary tract infection
Source: Microbiol Spectr. 2026 May 29;14(7):e03397-25. doi: 10.1128/spectrum.03397-25 (PMC13340212; doi:10.1128/spectrum.03397-25)
Supplement: Supplemental material — Fig. S1 to S3; Tables S1 to S4. [file spectrum.03397-25-s0001.pdf]

SUPPLEMENTARY FIGURES AND TABLES

Harnessing Lytic Phages for Biofilm Control in Carbapenem-Resistant *Klebsiella pneumoniae* Causing Urinary Tract Infection

***Dipendra Kumar Mandal**[1,2,3], Elisha Upadhaya[1], Puja Dahal[1], Gaurav Adhikari[1], Rojina Pandey[1], Abdul Rehaman[1], Sudip Timilsina[1], Pragya Sapkota[1], Shobha Amagain[1], David Pun[1], Kundan Khadka[1], Keshab Gorathoki[1], Bijita Neupane[1], Sangharsika Chaudhary[1], Sushila Thapa[1], Binod Khadka[4], Gun Raj Dhungana[5], Gorkha Raj Giri[1], Pragati Pradhan[1], Krishna Das Manandhar[1], Roshan Nepal[6], Rajindra Napit[1,7], Rajani Malla[1]

Author affiliations

1. Central Department of Biotechnology, Tribhuvan University, Kirtipur, Kathmandu, Nepal
2. Manmohan Memorial Institute of Health Sciences, Soalteemode, Kathmandu, Nepal
3. Provincial Public Health Laboratory, Lumbini Province, Nepal
4. Center for Molecular Dynamics, Nepal
5. Department of ODS and Research, Meharry Medical College, Nashville, Tennessee, USA
6. Commonwealth Scientific and Industrial Research Organization (CSIRO), Hobart, Tas 7004, Australia
7. School of Medicine, Deakin University, Australia

***Corresponding author**

Dipendra Kumar Mandal

Central Department of Biotechnology, Tribhuvan University

Email: dipendradas@iom.edu.np

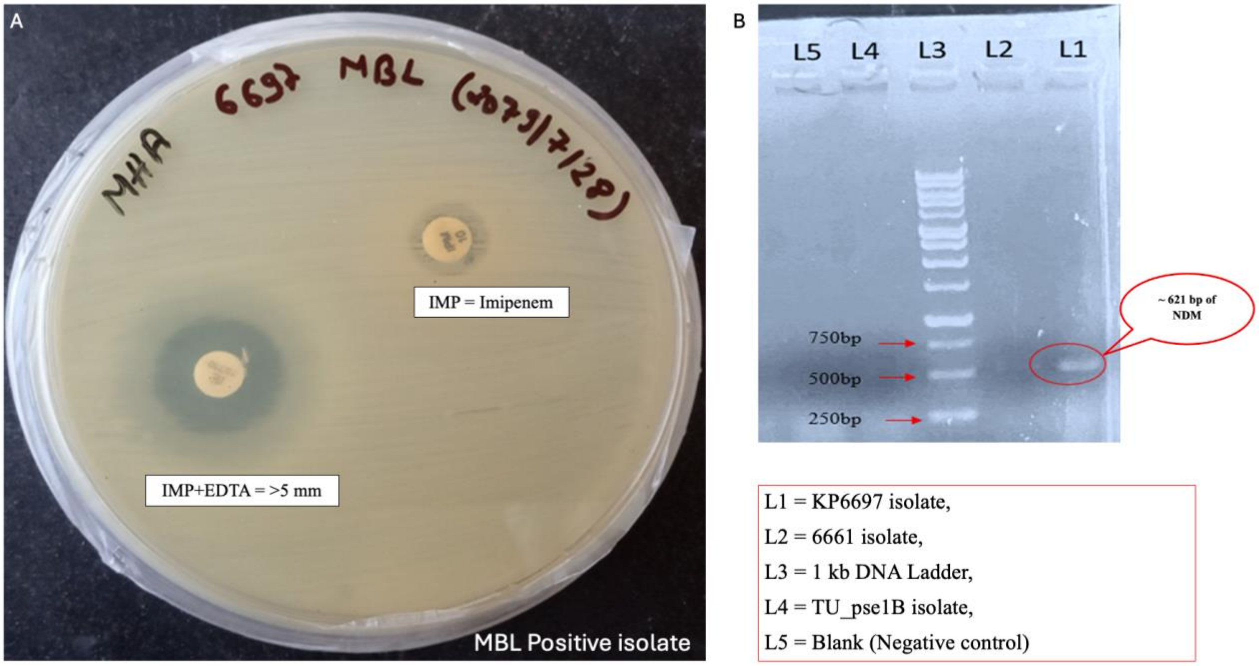

Fig. S1: Detection of Metallo-β-lactamase (MBL) by combined disk diffusion (A) and PCR method (B)

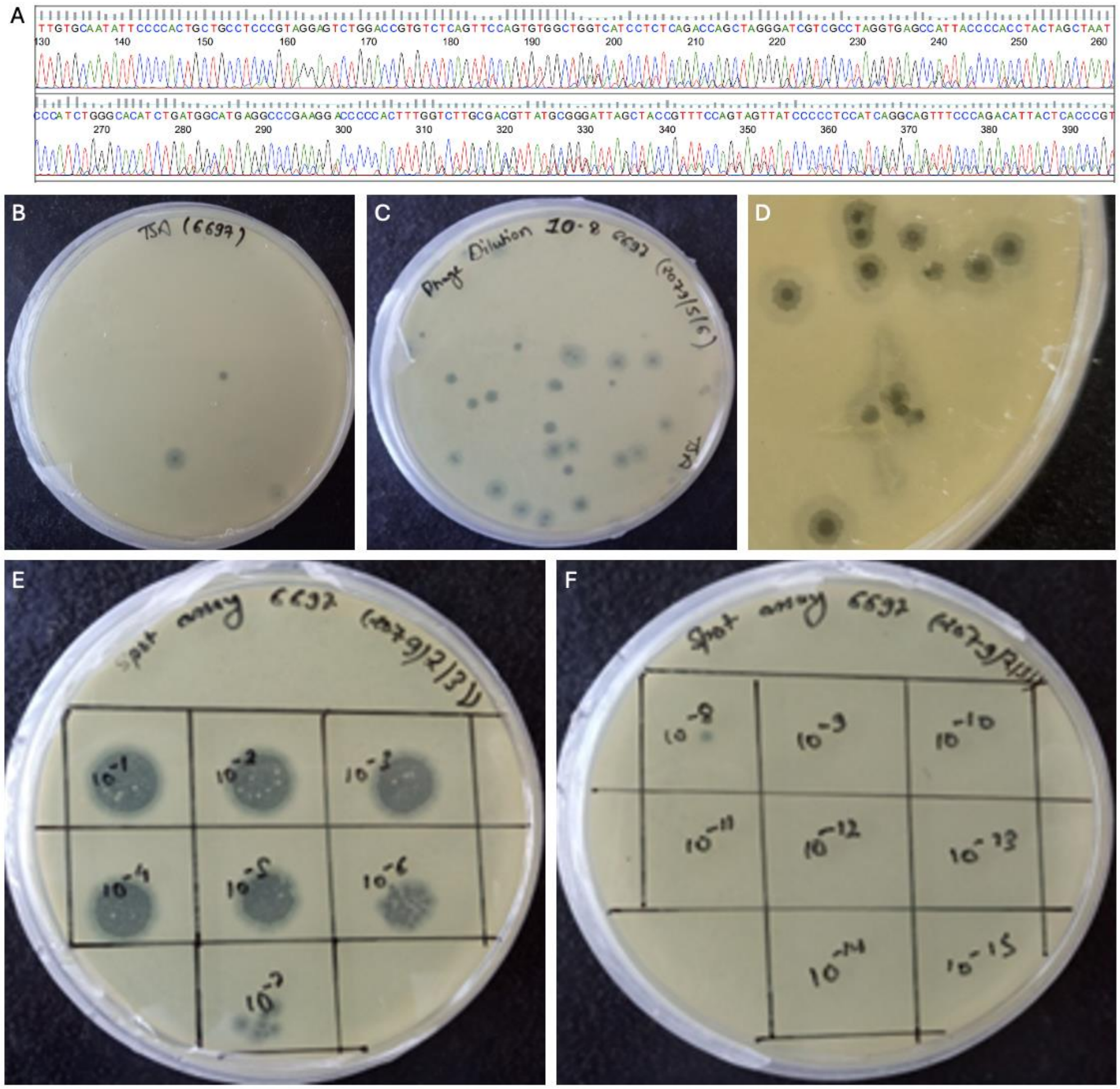

Fig. S2: Phage isolation and plaque morphology at various dilution.

A) Chromatogram pf 16S rRNA gene sequencing for verification of *Klebsiella pneumoniae* isolate.
B) Initial plate of bacteriophage isolation directly from water sample.
C) 10^{-8} dilution showing countable plaques of isolated bacteriophage.
D) Plaque morphology after purification.
E) Spot assay for phage titration (dilution 10^{-1} to 10^{-7}).
F) Spot assay for phage titration (dilution 10^{-8} to 10^{-15}).

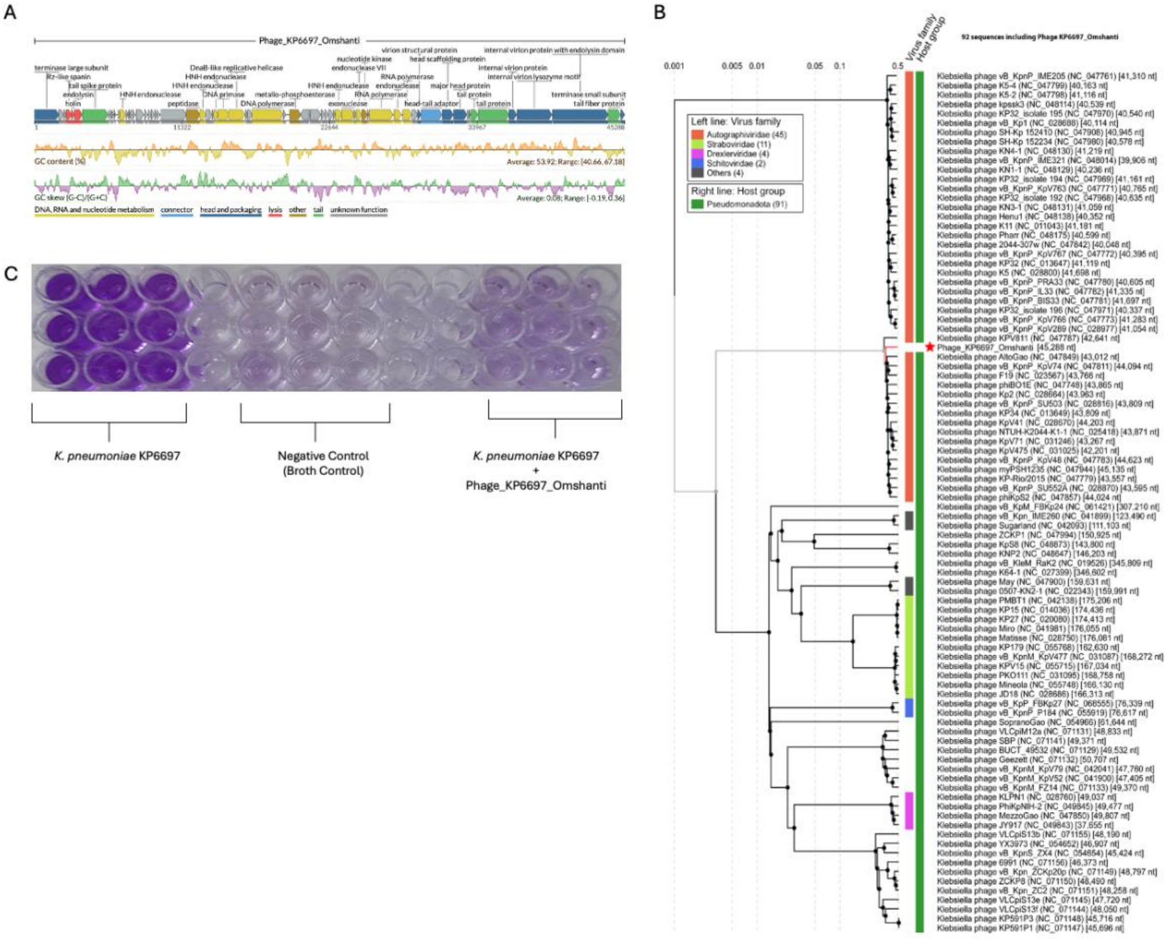

Fig. S3: Genome annotation of Phage_KP6697_Omshanti.

A) The genomic mapping of the Phage_KP6697_Omshanti with functional annotation and GC content based on Pharokka output and LoVis4u output.

B) taxonomic inference via phylogenetic analysis against all the Klebsiella phages available at Virus-Host DB. The tree was generated using ViPTree webtool.

C) Representative crystal violet (CV) assay.

Table S1: List of metabolic pathways detected with numbers of associated genes in *K. pneumoniae* (KP6697) isolate and their respective KO* numbers.

ko00001	KEGG Orthology (KO) (2685)
**Protein families:**	**Metabolism**
ko01000	Enzymes (1235)
ko01001	Protein kinases (27)
ko01009	Protein phosphatases and associated proteins (4)
ko01002	Peptidases and inhibitors (82)
ko01003	Glycosyltransferases (11)
ko01005	Lipopolysaccharide biosynthesis proteins-22
ko01011	Peptidoglycan biosynthesis and degradation proteins (46)
ko01004	Lipid biosynthesis proteins (18)
ko01008	Polyketide biosynthesis proteins (4)
ko01006	Prenyltransferases (7)
ko01007	Amino acid related enzymes (42)
ko00194	Photosynthesis proteins (9)
**Protein families:**	**Genetic information processing**
ko03000	Transcription factors (154)
ko03021	Transcription machinery (21)
ko03019	Messenger RNA biogenesis (27)
ko03011	Ribosome (47)
ko03009	Ribosome biogenesis (68)
ko03016	Transfer RNA biogenesis (89)
ko03012	Translation factors (19)
ko03110	Chaperones and folding catalysts (49)
ko04131	Membrane trafficking (5)
ko03051	Proteasome (1)
ko03032	DNA replication proteins (33)
ko03036	Chromosome and associated proteins (57)
ko03400	DNA repair and recombination proteins (90)
ko03029	Mitochondrial biogenesis (24)
**Protein families:**	**Signaling and cellular processes**
ko02000	Transporters (536)
ko02044	Secretion system (68)
ko02042	Bacterial toxins (2)
ko02022	Two-component system (53)
ko02035	Bacterial motility proteins (9)
ko04812	Cytoskeleton proteins (7)
ko04147	Exosome (37)
ko02048	Prokaryotic defense system (30)
ko01504	Antimicrobial resistance genes (22)
ko00537	Glycosylphosphatidylinositol (GPI)-anchored proteins (3)

* KO = KEGG (Kyoto Encyclopedia of Genes and Genomes) orthology

Table S2: List of social genes found in the host isolate *Klebsiella pneumoniae* KP6697, their KO* identification number and definitions.

KO numbers	KO definition-extracellular action	KO numbers	KO definition-Intracellular action
**K02507**	protein transport protein HofQ	K25706	tRNA N6-adenosine threonylcarbamoyltransferase [EC:2.3.1.234]
**K03092**	RNA polymerase sigma-54 factor	K03814	monofunctional glycosyltransferase [EC:2.4.99.28]
**K03086**	RNA polymerase primary sigma factor	K07259	serine-type D-Ala-D-Ala carboxypeptidase/endopeptidase (penicillin-binding protein 4) [EC:3.4.16.4 3.4.21.-]
**K04333**	LuxR family transcriptional regulator, csgAB operon transcriptional regulatory protein	K07348	minor fimbrial subunit
**K07713**	two-component system, NtrC family, response regulator HydG	K07351	fimbrial protein
**K03087**	RNA polymerase nonessential primary-like sigma factor	K07345	major type 1 subunit fimbrin (pilin)
**K15836**	formate hydrogenlyase transcriptional activator	K07345	major type 1 subunit fimbrin (pilin)
**K12266**	anaerobic nitric oxide reductase transcription regulator	K07345	major type 1 subunit fimbrin (pilin)
**K07173**	S-ribosylhomocysteine lyase [EC:4.4.1.21]	K07345	major type 1 subunit fimbrin (pilin)
**K03695**	ATP-dependent Clp protease ATP-binding subunit ClpB	K07348	minor fimbrial subunit
**K07715**	two-component system, NtrC family, response regulator GlrR	K04564	superoxide dismutase, Fe-Mn family [EC:1.15.1.1]
**K07782**	LuxR family transcriptional regulator, quorum-sensing system regulator SdiA	K06113	arabinan endo-1,5-alpha-L-arabinosidase [EC:3.2.1.99]
**K07684**	two-component system, NarL family, nitrate/nitrite response regulator NarL	K04564	superoxide dismutase, Fe-Mn family [EC:1.15.1.1]
**K23188**	iron-siderophore transport system ATP-binding protein [EC:7.2.2.17 7.2.2.-]	K03570	rod shape-determining protein MreC
**K23186**	iron-siderophore transport system permease protein	K07121	uncharacterized protein
**K25111**	iron-siderophore transport system permease protein	K07350	minor fimbrial subunit
**K07659**	two-component system, OmpR family, phosphate regulon response regulator OmpR	K16077	sucrose porin
**K06998**	trans-2,3-dihydro-3-hydroxyanthranilate isomerase [EC:5.3.3.17]	K01649	2-isopropylmalate synthase [EC:2.3.3.13]
**K18699**	beta-lactamase class A SHV [EC:3.5.2.6]	K07345	major type 1 subunit fimbrin (pilin)
**K11907**	type VI secretion system protein VasG	K01423	beta-barrel assembly-enhancing protease [EC:3.4.-.-]
**K16090**	catecholate siderophore receptor	NA	NA
**K01252**	bifunctional isochorismate lyase / aryl carrier protein [EC:3.3.2.1 6.3.2.14]	K01489	cytidine deaminase [EC:3.5.4.5]
**K08225**	MFS transporter, ENTS family, enterobactin (siderophore) exporter	K07289	AsmA protein
**K23186**	iron-siderophore transport system permease protein	K06192	paraquat-inducible protein B
**K23187**	iron-siderophore transport system permease protein	K07001	NTE family protein
**K23188**	iron-siderophore transport system ATP-binding protein [EC:7.2.2.17 7.2.2.-]	K06151	gluconate 2-dehydrogenase alpha chain [EC:1.1.99.3]
**K07657**	two-component system, OmpR family, phosphate regulon response regulator PhoB	K03615	H+/Na+-translocating ferredoxin:NAD+ oxidoreductase subunit C [EC:7.1.1.11 7.2.1.2]
**K18988**	serine-type D-Ala-D-Ala carboxypeptidase/endopeptidase [EC:3.4.16.4 3.4.21.-]	K24847	23S rRNA 5-hydroxycytidine C2501 synthase
**K04772**	serine protease DegQ [EC:3.4.21.-]	K07345	major type 1 subunit fimbrin (pilin)
**K10829**	ferric hydroxamate transport system ATP-binding protein [EC:7.2.2.16]	K21948	3-dehydrotetronate 4-kinase [EC:2.7.1.217]
**K02465**	general secretion pathway protein S	K04775	protease YdgD [EC:3.4.21.-]
**K02451**	general secretion pathway protein B	K13650	MqsR-controlled colanic acid and biofilm protein A
**K02452**	general secretion pathway protein C	K03737	pyruvate-ferredoxin/flavodoxin oxidoreductase [EC:1.2.7.1 1.2.7.-]
**K02453**	general secretion pathway protein D	K07146	UPF0176 protein
**K02454**	general secretion pathway protein E [EC:7.4.2.8]	K00800	3-phosphoshikimate 1-carboxyvinyltransferase [EC:2.5.1.19]
**K02455**	general secretion pathway protein F	K09136	ribosomal protein S12 methylthiotransferase accessory factor
**K02456**	general secretion pathway protein G	K00849	galactokinase [EC:2.7.1.6]
**K02457**	general secretion pathway protein H	K01835	phosphoglucomutase [EC:5.4.2.2]
**K02458**	general secretion pathway protein I	K03642	peptidoglycan lytic transglycosylase [EC:4.2.2.29]
**K02459**	general secretion pathway protein J	K02364	L-serine---[L-seryl-carrier protein] ligase [EC:6.3.2.14 6.2.1.72]
**K02460**	general secretion pathway protein K	K16076	outer membrane porin protein LC
**K02461**	general secretion pathway protein L	K00892	inosine kinase [EC:2.7.1.73]
**K02462**	general secretion pathway protein M	K03546	DNA repair protein SbcC/Rad50
**K02463**	general secretion pathway protein N	K21967	Mat/Ecp fimbriae adhesin
**K02464**	general secretion pathway protein O [EC:3.4.23.43 2.1.1.-]	K01200	pullulanase [EC:3.2.1.41]
**K21478**	poly-beta-1,6-N-acetyl-D-glucosamine N-deacetylase [EC:3.5.1.-]	K20470	trehalose monomycolate/heme transporter
**K03807**	AmpE protein	K12524	bifunctional aspartokinase / homoserine dehydrogenase 1 [EC:2.7.2.4 1.1.1.3]
**K03806**	N-acetyl-anhydromuramoyl-L-alanine amidase [EC:3.5.1.28]	K01198	xylan 1,4-beta-xylosidase [EC:3.2.1.37]

**K02682**	prepilin peptidase dependent protein D	NA	NA
**K02504**	protein transport protein HofB	K01448	N-acetylmuramoyl-L-alanine amidase [EC:3.5.1.28]
**K02505**	protein transport protein HofC	NA	NA
**K03590**	cell division protein FtsA	K07345	major type 1 subunit fimbrin (pilin)
**K11935**	biofilm PGA synthesis protein PgaA	K00928	aspartate kinase [EC:2.7.2.4]
**K11931**	poly-beta-1,6-N-acetyl-D-glucosamine N-deacetylase [EC:3.5.1.-]	K00848	rhamnulokinase [EC:2.7.1.5]
**K11937**	biofilm PGA synthesis protein PgaD	K03641	TolB protein
**K07713**	two-component system, NtrC family, response regulator HydG	NA	NA
**K07662**	two-component system, OmpR family, response regulator CpxR	NA	NA
**K07712**	two-component system, NtrC family, nitrogen regulation response regulator GlnG	NA	NA

* KO = KEGG (Kyoto Encyclopedia of Genes and Genomes) orthology

Table S3: Prophages found in *Klebsiella pneumoniae* KP6697 isolate and their features.

**Prophage code**	**153**	**267**	**349**	**529**	**1172**
Length (in base pairs)	425,528	40,614	53,577	35,094	39,201
Guanine-cytosine (G+C) content	58.42 %	51.07 %	53.09 %	51.77 %	50.16 %
tRNAs	2	0	0	0	0
Average gene size (in bp)	414	55	56	53	41
Completeness (based on CheckV)	High-quality	High-quality	High-quality	Medium-quality	High-quality
Lifestyle	Temperate	Temperate	Temperate	Temperate	Temperate
Predicted bacteria host	*K. pneumoniae*	*K. pneumoniae*	*K. pneumoniae*	*E. coli*	*K. pneumoniae*
Predicted taxonomic class		*Caudoviricetes*	*Caudoviricetes*	*Caudoviricetes*	*Caudoviricetes*
**Protein features**					
Hypothetical proteins	4	10	10	9	6
Assembly gene	18	6	2	2	4
Infection gene	22	3	8	5	8
Assembly & infection gene	5	2	5	9	3
Lysis gene	20	4	1	0	4
Lysis, assembly & infection gene	1	0	0	0	0
Lysis & infection gene	1	0	0	0	0
Lysis & regulation gene	1	0	0	0	0
Lysis & replication gene	1	0	0	0	0
Lysis & assembly gene	3	0	0	0	0
Immune gene	13	0	0	0	2
Replication & packaging gene	1	1	0	0	0
Replication & regulation gene	3	3	1	0	0
Regulation gene	32	2	3	2	2
Regulation & assembly gene	2	0	0	0	0
Regulation, assembly & immune gene	3	0	0	0	0
Regulation & immune gene	1	0	0	0	0
Replication gene	37	1	5	1	1
Replication & immune gene	1	0	0	0	0
Replication & infection gene	3	0	0	0	0
Replication & assembly gene	2	0	0	0	0
Replication, regulation & assembly gene	1	0	0	0	0
Replication & tRNAgene	1	0	0	0	0
Replication & tRNA regulation gene	1	0	0	0	0
Replication, regulation & immune gene	1	0	0	0	0
tRNA & infection	1	0	0	0	0
Integration & assembly gene	2	0	0	0	0
integration, replication, regulation & packaging gene	3	0	0	0	0
Integration gene	11	1	0	2	2
Integration & replication gene	0	1	0	0	0
Packaging gene	5	2	6	2	3
Packaging & assembly gene	0	1	0	0	0
Virulence factors (VFDB database)	22	0	0	0	0
AMR genes (CARD database)	5	0	0	0	0

Table S4: Sequences producing significant alignments with Phage_KP6697_Omshanti in NCBI database based on blastn (as of 07-Apr-2026)

SN	Scientific Name (NCBI)	Max Score	Total Score	Query Cover	E value	Per. ident	Acc. Len	Accession
1	Klebsiella phage RCIP0082	18173	49693	81%	0	93.66	44678	OR532876.1
2	Klebsiella phage vB_Kpn_HF0522	17813	46439	78%	0	92.41	42437	PP836776.1
3	Klebsiella phage RCIP0111	17756	46426	76%	0	93.06	43570	OR532905.1
4	Klebsiella phage P6a	17666	47011	77%	0	92.94	43717	PQ133617.1
5	Klebsiella phage KP-PHA-Meh	17588	45671	76%	0	92.82	42649	OR982685.1
6	Klebsiella phage vB_KpnP_W17	17571	44845	76%	0	92.79	44237	PP728208.1
7	Klebsiella phage VLCpiA1k	17459	46404	75%	0	92.64	43278	ON602739.1
8	Klebsiella pneumoniae phage	17398	47446	78%	0	92.55	44964	OU509534.1
9	Klebsiella phage RSU-F1K2	17398	43853	75%	0	92.54	43675	PX670392.1
10	Klebsiella phage RSU-F1K1	17398	43840	75%	0	92.54	43673	PX670391.1
11	Klebsiella phage phi1_146044	17392	46957	76%	0	92.53	44064	PP889493.1
12	Klebsiella phage 2024.TE.12363	17379	47197	77%	0	92.51	43072	PZ055532.1
13	Klebsiella phage P7285	17372	46076	75%	0	92.5	45092	PQ133621.1
14	Klebsiella phage RCIP0039	17102	42024	71%	0	91.39	42926	OR532833.1
15	Klebsiella phage P1010	17012	49073	81%	0	91.97	44270	ON132389.1
16	Klebsiella phage phi1_066012	17012	47633	78%	0	91.96	44294	PP889456.1
17	Klebsiella phage phi1_066009	17012	47627	78%	0	91.96	44293	PP889453.1
18	Klebsiella phage phi1_066010	17012	47633	78%	0	91.96	44294	PP889454.1
19	Klebsiella phage vB_KpnP_IME309	17005	48010	79%	0	93.45	43286	PQ998984.1
20	Klebsiella phage phi1_066011	17003	47600	78%	0	91.94	44292	PP889455.1
21	Klebsiella phage phi1_146033	16801	45478	77%	0	93.1	44491	PP889483.1
22	Klebsiella phage Henu2_3	16476	43667	74%	0	91.17	42878	PQ394119.1
23	Klebsiella phage KA	16449	48720	77%	0	93.09	49319	OR809313.1
24	Klebsiella phage KpV71	16427	46136	77%	0	91.1	43267	NC_031246.1
25	Klebsiella phage vB_KpnP_IME337	16364	45216	80%	0	91.01	44266	MN176573.1
26	Klebsiella phage vB_KpnP_GZMU_VR401	16323	47890	79%	0	90.97	42944	PV253913.1
27	Klebsiella phage Putnam	16312	45937	79%	0	90.95	43554	PQ358941.1
28	Klebsiella phage KP-Rio/2015	16288	44231	77%	0	90.91	43557	NC_047779.1
29	Klebsiella phage VLCpiA1o	16286	46331	78%	0	90.91	42793	ON602724.1
30	Klebsiella phage phiBO1E	16277	44836	76%	0	90.86	43865	NC_047748.1
31	Klebsiella phage vB_KpnP_SU552A	16271	47705	79%	0	90.89	43595	NC_028870.1
32	Klebsiella phage vB_KpnA_SCNJ1-Z	16253	45166	78%	0	90.86	43428	OQ689084.1
33	Klebsiella phage RSU-F6K4	16244	41464	74%	0	90.81	44444	PX670401.1
34	Klebsiella phage VLCpiA1g	16236	46956	80%	0	90.83	44132	ON602761.1
35	Klebsiella phage KpV475	16196	46567	79%	0	90.77	42201	NC_031025.1
36	Klebsiella phage 2024.TE.12369	16194	46131	80%	0	90.74	43914	PZ055535.1
37	Klebsiella phage vB_Kpn_CuaREV2	16175	45032	78%	0	90.75	44284	PX060903.1
38	Klebsiella phage JKP2	16150	48471	80%	0	92.12	43211	ON165415.1
39	Klebsiella phage 6993	16135	46805	80%	0	90.68	44351	OL362278.1
40	Klebsiella phage RSU-F4K4-1	16111	42951	77%	0	90.61	44307	PX670397.1
41	Klebsiella phage KPN234	16103	45612	77%	0	90.08	44193	PV446873.1
42	Klebsiella phage P252	16030	40272	70%	0	90.56	43944	PP934443.1
43	Klebsiella phage RSU-phaL3-2	16020	45788	77%	0	90.52	44164	PX670411.1
44	Klebsiella phage 6995	16015	46610	77%	0	90.51	42528	OL362279.1
45	Klebsiella virus KpV2883	15998	44983	78%	0	90.49	43508	MT682065.1
46	Klebsiella phage RCIP0044	15991	46297	76%	0	90.46	43541	OR532838.1
47	Klebsiella phage HenuGS	15961	48365	81%	0	90.43	44399	PQ362313.1
48	Klebsiella phage VKV295	15961	45312	77%	0	90.43	42380	OR287807.1
49	Klebsiella phage vB_KpnP_Bp5	15939	46009	77%	0	90.39	43872	MN116494.1
50	Klebsiella phage vB_KpP_AttikonH2	15910	46264	78%	0	90.35	44403	PP978610.1

- THE END -
